# Smartphones, social Media and Adolescent mental well-being: the impact of school policies Restricting dayTime use—protocol for a natural experimental observational study using mixed methods at secondary schools in England (SMART Schools Study)

**DOI:** 10.1136/bmjopen-2023-075832

**Published:** 2023-07-05

**Authors:** Grace Wood, Victoria Goodyear, Peymane Adab, Hareth Al-Janabi, Sally Fenton, Kirsty Jones, Maria Michail, Breanna Morrison, Paul Patterson, Alice J Sitch, Matthew Wade, Miranda Pallan

**Affiliations:** 1 School of Sport, Exercise and Rehabilitation Sciences, University of Birmingham, Birmingham, UK; 2 Institute for Mental Health, University of Birmingham, Birmingham, UK; 3 Institute of Applied Health Research, University of Birmingham, Birmingham, UK; 4 Head of School Support, Services for Education, Birmingham, UK; 5 School of Psychology, University of Birmingham, Birmingham, UK; 6 Birmingham Women’s and Children’s NHS Foundation Trust, Birmingham, UK; 7 NIHR Birmingham Biomedical Research Centre, University Hospitals Birmingham NHS Foundation Trust and University of Birmingham, Institute of Translational Medicine, Birmingham, UK; 8 ukactive Research Institute, London, UK; 9 Advanced Wellbeing Research Centre, Sheffield Hallam University College of Health Wellbeing and Life Sciences, Sheffield, UK

**Keywords:** adolescent, mental health, schools

## Abstract

**Introduction:**

Smartphone and social media use is prevalent during adolescence, with high levels of use associated with lower levels of mental well-being. Secondary schools in the UK have introduced policies that restrict daytime use of smartphones and social media, but there is no evaluation on the impact of these policies on adolescent mental well-being. The SMART Schools Study aims to determine the impact of daytime restrictions of smartphone and social media use on indicators of adolescent mental well-being, anxiety, depression, physical activity, sleep, classroom behaviour, attainment and addictive social media use.

**Methods and analysis:**

This is a natural experimental observational study using mixed methods. Secondary schools within a 100 mile radius of the recruiting centre in the West Midlands (UK) have been categorised into two groups: Schools that restrict (intervention) and permit (comparator) daytime use of smartphones. We aim to recruit 30 schools (20 restrictive, 10 permissive) and 1170 pupils aged 12–13 and 14–15 years. We will collect data on mental well-being, anxiety and depressive symptoms, phone and social media use, sleep and physical activity from pupil surveys, and accelerometers. Policy implementation measures and data on individual pupil factors will be collected through school staff surveys, and website/policy analysis. Six case study schools will explore individual, school and family/home factors that influence relationships between school smartphone policies, smartphone/social media use, and mental well-being. Economic evaluation will be completed through a cost–consequence analysis from an education sector perspective.

**Ethics and dissemination:**

Ethical approval was obtained from the University of Birmingham’s Research Ethics Committee (ERN_22-0723). Parents/carers of pupil participants can complete a form to opt their child out of the study. Pupil, school staff and parent/carer participants are asked to complete online/written consent (or assent). Findings will be disseminated through policy briefings, resources for schools, social media, reports, and open access publications.

**Trial registration number:**

ISRCTN77948572.

**Strengths and limitations of this study**

- This is a natural experimental observational study exploring the impact of school phone policies that restrict daytime use of smartphones and social media on adolescent mental well-being and associated behaviours, and the cost implications of these policies from an education sector perspective.
- Pupils will self-report data on time spent on their smartphones and social media by using data from their iOS or Android apps.
- Pupil outcomes will be collected from an online survey that includes validated measures for mental well-being, anxiety and depressive symptoms, addictive social media use, motives for social media use, health-related quality of life and demographic data; and from accelerometers measuring physical activity and sleep.
- Qualitative research will explore how individual, school and family/home factors influence relationships between school phone policies, phone/media use and mental well-being.
- This is a cross-sectional study and is limited to data collected in England.

## Introduction

Globally, mental disorders (eg, anxiety and depression) are the leading cause of disability in adolescents (age range: 10–19).^1 2^ In the UK, one fifth (20%) of adolescents are reported to have a mental health disorder,^3^ mostly anxiety and depression.^2^ Half of all mental health disorders start before the age of 14,^4 5^ and if left untreated, mental health problems are highly likely to persist well into adulthood.^2 6^ Poor mental well-being also negatively affects other aspects of adolescents’ lives, including cognitive, social and physical behaviours.^6 7^ For example, poor mental well-being is associated with higher rates of disruptive behaviour, school absence and lower educational attainment.^8 9^ Sleep problems are also common among adolescents diagnosed with anxiety and depression, and evidence suggests a bidirectional relationship between sleep disturbances and mental health problems.^10^ Levels of physical activity also decline significantly during adolescence, and this coincides with increased onset of mental health problems.^4 11^ Hence, adolescence is a crucial period for mental health interventions,^7^ and there is a pressing need to improve and develop approaches to mental health prevention and intervention.^6^

Smartphone and social media use is prevalent during adolescence, and accounts for the majority of their overall screen time.^12 13^ In the UK, most adolescents (98%) own a smartphone, and are reported to be active users of social media (93%),^14^ with comparable trends reported in other Western populations.^15^ Samples in the USA and the UK (2021–2022) estimate that the time adolescents spend on smartphones and social media ranges between one and a half hours and eight and a half hours per day,^12–14 16 17^ with most adolescents spending between 1 and 3 hours per day.^14 16 17^ Problematic social media use is also prevalent, with 12% of adolescents in England reported to exhibit addictive use behaviours.^18^

In moderation, smartphone and social media use (eg, <2 hours per day) can be advantageous for mental well-being and mental health,^19–24^ as well as other associated health and behavioural outcomes (eg, sleep, physical activity, classroom behaviour and attainment). However, at higher levels of use, the reverse effect tends to be seen, with increasing time spent on smartphones and social media associated with decreasing levels of mental well-being and higher levels of anxiety and depression.^17 19–21 23 25 26^ Poor academic performance, disruptive classroom behaviour and less time spent in physical activity and sleep are also more likely in adolescents who spend a greater proportion of time on smartphones and/or social media.^19–23 25 26^ Reducing the time adolescents spend on smartphones and social media is thus a plausible intervention to improve mental well-being, possibly operating through improving the related behavioural outcomes (eg, physical activity, sleep, academic performance, classroom behaviour). However, uncertainties in the strength of associations between smartphone/social media and mental well-being exist, and this is mainly due to reliance on self-reported use.^15 27–29^ Furthermore, individual (eg, gender, age, socioeconomic status)^13 16 30–33^ and family/home (eg, parental usage and attitudes toward technology)^34–37^ factors are also likely to impact on relationships between smartphone/social media use and mental well-being.

There is considerable evidence that school-based interventions can have beneficial effects on adolescent mental well-being and associated behavioural outcomes (eg, sleep, physical activity, classroom behaviour and attainment).^38–41^ Whole-school environment interventions that promote lifestyles conducive to good health are reported to have a more pronounced effect on mental well-being than individual approaches targeting knowledge and beliefs.^38 42^ A whole-school approach targets physical and social influences of health, and through the alignment of school policies, values and practices with effective school leadership, the whole school approach seeks to promote a set of values, attitudes and behaviours that encourage the development and maintenance of positive physical, social, cognitive and emotional habits.^38 40^ Evidence suggests that whole school policies related to health and well-being can: (1) reduce overall screen time; (2) positively influence mental well-being; and (3) improve physical activity, sleep, educational attainment and reduce disruptive classroom behaviour.^43–45^ Therefore, whole school policies aiming to influence smartphone and social media use have the potential to positively impact on adolescents’ mental well-being.

School phone policies that restrict daytime phone/media use are an example of a current whole-school environment intervention. In the UK, Australia, Sweden, Czech Republic and elsewhere, many schools have introduced school policies that restrict daytime use of smartphones in order to reduce classroom disruptive behaviour and cyberbullying incidents and improve attainment.^44 46 47^ We suggest that these policies have the potential to lower the overall time adolescents spend on the smartphones/social media, which may improve mental well-being and associated behavioural outcomes. However, there is currently no evaluation of the effect of school smartphone policies on mental well-being and there is limited evidence on how smartphone policies are implemented in schools.^48^

The SMART Schools Study aims to determine the impact of school daytime restrictions of smartphone and social media use on adolescent mental well-being (primary outcome), anxiety, depression, physical activity, sleep, classroom behaviour, attainment and addictive social media use. We will do this by comparing impacts in two different secondary school phone policy contexts: (1) schools that do not permit smartphone use during recreational time in the school day (intervention); and (2) schools that permit smartphone use during recreational time (breaks/lunchtimes) (comparator). We will also explore how variation in school-based, individual and family/home factors influences the relationship between school phone policies, smartphone and social media use and mental well-being. We will conduct an economic evaluation in the form of a cost–consequence analysis from an education sector perspective.

## Methods and analysis

This is a natural experimental observational study using mixed methods, taking place between April 2022 and July 2024. Quantitative and qualitative data will be collected from all schools in the sample (n=30) to compare outcomes between restrictive (intervention) and permissive (comparator) school policies and to complete an economic evaluation. Qualitative data will be collected from six case study schools to understand the contextual factors that could influence relationships between school policies, smartphone and social media use and mental well-being.

### Intervention: school smartphone policies that restrict daytime use

**Figure 1:**
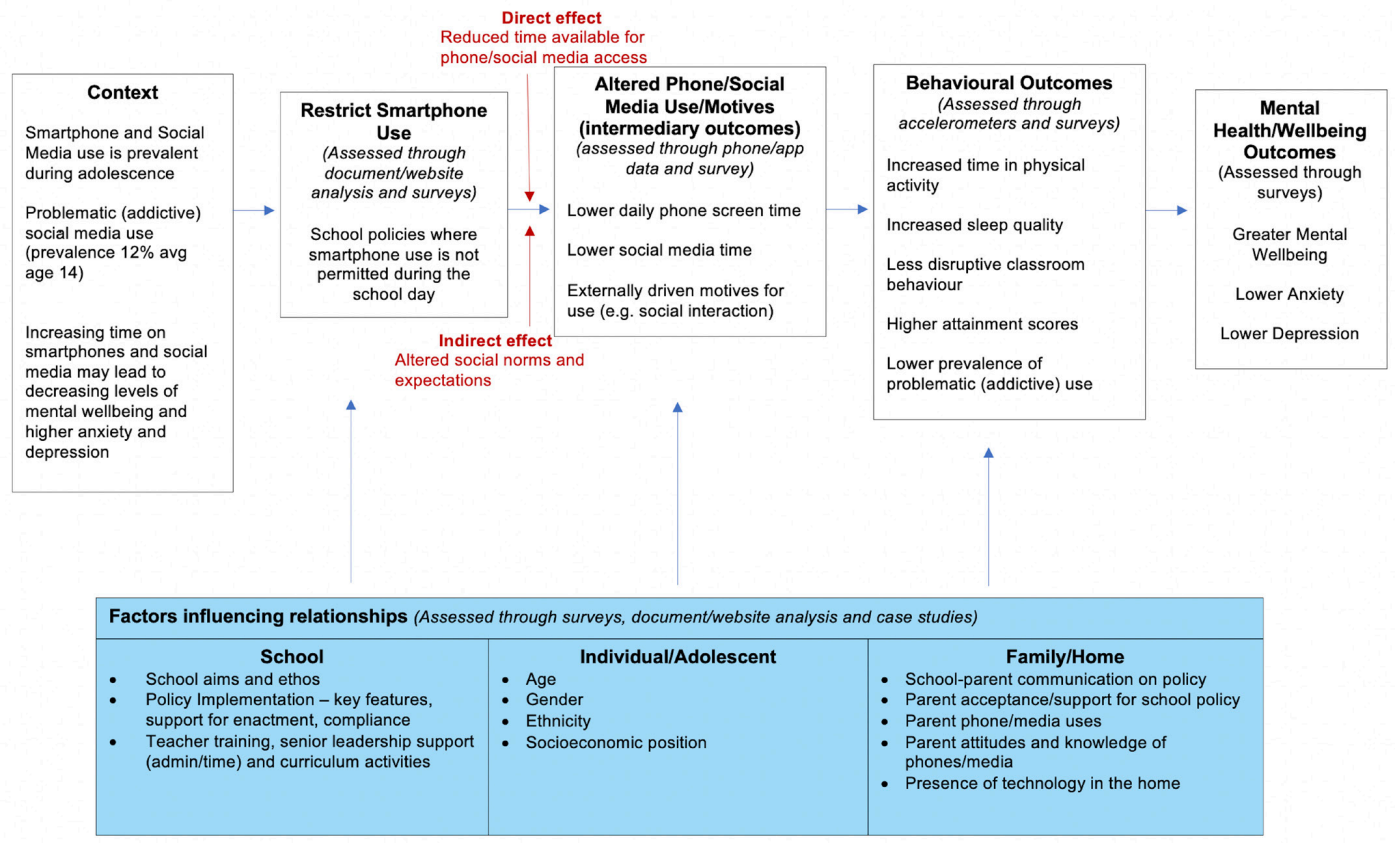
Logic model and theory of change for the influence of school policies that restrict daytime use of smartphones on mental well-being and other associated behavioural and health outcomes in adolescents.

The intervention and data capture are directed by our logic model (figure 1) which integrates multiple theories and evidence. First, we adhere to displacement theories^19^ to propose that reducing the time adolescents spend on phones/media (ie, restricting school time use) is optimal for mental well-being. Overuse can displace other mental well-being promoting activities (eg, sleep and physical activity) and very low use can deprive adolescents of interactions that support mental well-being (eg, affect and relationships).^19 21 23^ Second, psychological motives drive phone/media use; motives related to enhancement and social interactions promote mental well-being and motives related to coping and conformity (eg, Fear of Missing Out) are associated with problematic use (addiction) and poor mental well-being.^49 50^ Hence the school policy and ethos have the potential to influence adolescents’ motives for using phones/media, which may impact on mental well-being. Third, the ecological model of social influence proposes three agents that shape well-being and technology use, including the school environment, home/family and individual factors.^51^ Finally, policy enactment and implementation process models^39 52 53^ identify that school policy implementation effects will be shaped by social processes (eg, training, leadership, compliance, administration, family-–school interactions).

Informed by our patient and public involvement (PPI) activities and school smartphone policy analysis, the components of the intervention (ie, restrictive school smartphone policies) are presented in table 1. In table 2 we have outlined variations in school smartphone policies and our classification of these variations into two school policy groups: restrictive (intervention) and permissive (comparator).

**Table 1:** Components of the intervention, guided by the template for intervention description and replication (TiDieR) checklist^75^

TiDieR components	SMART Schools intervention
Description	School policy prohibiting the use of smartphones during the school day
Materials	The policy may be communicated to parents/carers and adolescents in a variety of ways, such as through school information packs, assemblies, letters and/or the school website
Procedures	Adolescents are not permitted to use their smartphones during lessons or recreational time in the school day, and their smartphones must not be seen in school during these times
Provider	Schools (or MATs*) develop their own policies, often in consultation with school staff, parents and/or school governors and in relation to the school ethos
Mode of delivery	The SLT and school staff enact the school policy and are required to administer behavioural consequences for adolescents who use their smartphone during the day, such as smartphone confiscation, detention and/or parent–school meeting
Time period	Schools vary in terms of how long their school smartphone has been implemented
Tailoring	Schools have developed policies according to their specific school contexts (or MATs*). Policies usually apply to the whole school, although in some schools sixth form pupils (age 16+) may be permitted to use their smartphones during the school day (this age group will not be investigated in this study)
Adherence and fidelity	The degree to which pupils and teachers adhere to the school policy, and parents/carers are in support of the policy varies across schools

*MATs: non-profit companies that manage more than one academy.^76^

MAT, multiacademy trusts; SLT, senior leadership team.

**Table 2:** Classifications of variations in school smartphone policies as restrictive (intervention) and permissive (comparator)

Restrictive school smartphone policies (intervention)	Permissive school smartphone policies (comparator)
Allow smartphones onto school premises but insist these are not to be used during the school day and are turned off and out of sight	Allow pupils to carry smartphones and use them at any time point during the day
Allow smartphones onto school premises, but only allow use if sanctioned by teaching staff for educational activities (eg, use of calculator)	Allow pupils to carry smartphones and use them at specific time points during the day (eg, breaks and lunch)
Allow smartphones onto premises but insist they are left in a specified place during the school day for example, school reception or lockers	Allow pupils to carry smartphones and use them for personal use with consent from school staff
Pupils are not allowed to carry their smartphones onto school premises at any time	Allow pupils to carry smartphones and use within designated areas or zones

### Study setting

The sampling frame comprises UK secondary schools (ages 11–19) located within a 100 mile radius of the recruiting centre in the West Midlands. Sixty-four local authorities are included in the sampling frame from the West Midlands, East Midlands, East, South East, South West and North West. The schools are situated in regions of high and low levels of deprivation and in areas that have high^54^ and low proportions of Black, Asian and Minority Ethnic (BAME) groups.^55 56^ Schools other than state-funded mainstream schools (special schools, pupil referral units and independent schools) were excluded because it was expected that there would be additional influences on mental well-being. Schools that did not have an accessible smartphone policy and/or had different smartphone policies for the year groups 8 and 10, and schools with missing data that were required for the propensity score estimation (see sampling and participants) were also excluded (n=10,810). A total of 1345 secondary schools are included in our sampling frame; 1220 schools with policies classified as restrictive (intervention) and 125 as permissive (comparator).

### Sampling and participants

To improve the comparability of the two school groups, stratified sampling based on propensity scores was employed.^57^ We obtained routine data from the Department for Education on the following school characteristics: region, school type, urban or rural, total pupil roll size, Income Deprivation Affecting Children Index (IDACI),^58^ inclusion of a sixth form, selective or non-selective admissions policy, religious affiliation and the proportion of pupils with the following characteristics: male, from BAME groups, English as an additional language, eligible for free school meals and special education needs. Propensity scores were calculated using restrictive or permissive school smartphone policies as the outcome and school characteristics as explanatory variables. Propensity score terciles were then used to create three groups with subsequent division by restrictive or permissive policy type, resulting in six distinct sampling groups. Schools in each group have been randomly ordered and are being invited sequentially to participate, aiming to recruit six to seven schools from each restrictive tercile and three to four from each permissive tercile to achieve a sample size of 20 schools with restrictive and 10 schools with permissive policies.

In participating schools, two classes of mixed ability year 8 (age 12–13) and year 10 (age 14–15) pupils are recruited. We are focusing on adolescents in this age range because of the age of onset and prevalence of mental illness,^5^ and the prevalence of smartphone and social media use by adolescents aged 12–15.^7 14 59^ Within this age range we may also be able to observe potential differences in smartphone/social media use relationships with well-being.^33^ For example, year 8 pupils are likely to be newer users of phones/media and physical activity levels begin to decline at this age, particularly among girls.^7 13^ In year 10, pupils are more likely to be established phone/media users, mental well-being tends to be lower, and this age group are approaching the peak onset of mental health conditions.^4^ In addition to adolescent participants, the form teacher for each class recruited (or an equivalent teacher responsible for the class) and a member of senior leadership team (SLT) who is responsible for the school smartphone policy are recruited from each school.

### Sample size calculation

To account for the imbalance of schools in our sample that have permissive (n=125) and restrictive policies (n=1220), we are recruiting schools using a 2:1 (restrictive: permissive) ratio. The primary outcome of mental well-being will be measured using the Warwick-Edinburgh Mental Well-Being Scale (WEMWBS; score range=14–70).^60^ To detect a mean difference in score of three points (considered the minimum clinically important difference^60^ between the two school groups), assuming a SD of 6.8^61^ and an ICC of 0.1 (a conservative estimate^62^), with 90% power and 5% significance, we require 20 schools in the restrictive and 10 schools in the permissive smartphone policy groups, with an average cluster size of 39 (1170 pupil participants in total; 780 in the restrictive, and 390 in the permissive policy groups).

In each participating class, we aim to recruit a minimum of 19–20 pupils (67% if estimated class size n=30). In studies with multiple layers of clustering (here classes within schools), it is conservative to treat clusters within clusters as one larger cluster, which is the approach used here.^63^

### Recruitment

School recruitment commenced in September 2022 and will continue until December 2023. A study advertisement has been emailed to all schools in the sampling frame. Following our propensity sampling approach, schools are then invited by post and email with a telephone follow-up. In participating schools, a School Liaison Member (SLM) of staff is identified and a school–university contract outlining expected commitments signed. Subsequently, a member of SLT responsible for the smartphone policy, and pupils and teachers from the year 8 and year 10 classes are recruited. £600 compensation is allocated to each school and a £5 voucher per pupil participant.

Parents/carers are provided with written detailed information about the study, what their child’s participation will involve and how their child’s data will be processed. Schools are asked to assist in the distribution of this information to parents/carers in different formats (eg, email, post, text messages, website, and so on).

### Data collection

Data collection methods include self-administered surveys for pupils, teachers and a member of the SLT; accelerometer measured physical activity and sleep for pupils; and document analysis. All online surveys are completed using university-approved online survey software (REDCap). Table 3 provides an overview of outcomes, measures and timepoints of evaluation.

**Table 3:** Overview of outcomes, measures and timepoints of evaluation

Outcome	Outcome measures	Timepoint(s) of evaluation
Primary outcome		
Mental well-being	Warwick-Edinburgh Mental Well-Being Scale (WEMWBS)^60^/pupil survey	Measured over the past 2 weeks at two time points, 4–8 weeks apart
Secondary outcomes		
Anxiety symptoms	Generalised Anxiety Disorder Assessment (GAD-7)^77^/pupil survey	One time point measured over the past 2 weeks
Depressive symptoms	Patient Health Questionnaire (PHQ-9)^78^/pupil survey	One time point measured over the past 2 weeks
Addictive use	Problematic Social Media use Scale^49 50^/pupil survey	One time point based on usual use
Sleep	Sleep quality (indicated by sleep duration and sleep efficiency/accelerometers) Time pupils go to sleep and wake time on a school day and a weekend day/pupil survey	Over 7 days/usual behaviours
Physical activity	Physical activity (total and moderate-to-vigorous intensity (MVPA)/accelerometers) Active travel and sports/activity club engagement/ pupil survey	Over 7 days/usual behaviours Usual behaviours
Attainment	Assessment of whether pupils are below, above or working at their target grade in English and Maths/teacher survey	One time point—most recent assessment
Disruptive classroom behaviour	Pupil Behaviour Questionnaire^79^/teacher survey	One time point—current assessment
Intermediate outcomes		
Smartphone use duration	3 measures: (i) within school; (ii) over 24-hour period on a school day; (iii) on a weekend day/data captured from iOS and Android smartphones and will be self-reported through the pupil survey	One time point
Social media use duration	3 measures: (i) within school; (ii) over 24-hour period on a school day; (iii) on a weekend day/data captured from iOS and Android smartphones and will be self-reported through the pupil survey	One time point
Motives for social media use	Social Media Motives Questionnaire/pupil survey	One time point measured over past 12 months
Policy implementation measures		
Intervention components (school level data): school timetable; school policies (smartphone, mental health, behaviour, e-safety): school smartphone policy details (eg, rules/key features; time period of implementation; policy communication and understanding; adherence and fidelity and policy rationale)	School policy documents School handbooks School website content SLT survey Teacher survey Pupil survey	One time point
Individual factors (pupils): age, gender, ethnicity, socioeconomic position, eligibility for Free School Meals and whether pupils have English as an additional language and/or special educational needs	Pupil survey Teacher survey	One time point
Contextual factors: school, individual, family/home	Focus groups with school staff, pupils and parents/carers of school pupils	One time point
Economic variables		
Teacher time spent managing policy Indirect time spent managing behavioural problems Other related costs	SLT survey Teacher survey	One time point
Health-related quality of life/quality adjusted life years	The Child Health Utility Instrument (CHU9D)^80^/pupil survey	One time point/current assessment

SLT, senior leadership team.

#### Pupil online survey

Pupils complete an online survey at one time point (in school time) that includes validated measures for mental well-being, anxiety and depressive symptoms, addictive social media use, motives for social media use, health-related quality of life and demographic variables (eg, age, gender) (table 3). Within the survey, pupils are asked to self-report data on their physical activity levels, sleep and phone/media use, and by using data from their iOS or Android apps, they additionally self-report data on time spent on their smartphone and social media apps (table 3). Within the survey, pupils are also asked to report on their knowledge and understanding of the school smartphone policy and compliance with the school smartphone policy. The survey is completed on encrypted tablets, using a portable Wi-Fi hub owned by the research team.

#### Secondary measure of mental well-being

A second online survey (in school time) to measure mental well-being (table 3) is completed 4–8 weeks after pupils have completed the initial online survey. The SLM is sent an email link for pupils to complete.

#### Teacher online survey

Data are collected from the form teacher (or an equivalent teacher responsible for the class) for each participating pupil on: pupil attainment; classroom behaviour; and whether pupils are eligible for free school meals, have a special educational need or have English as an additional language (table 3). Within the survey, teachers are also asked to report on their knowledge and understanding of the school smartphone policy, compliance with the school smartphone policy and to report on the time they spend implementing the school smartphone policy (table 3). Teachers are sent the online survey by email.

#### SLT online survey

The SLT member responsible for the school smartphone policy is asked to complete survey questions on the SLT member’s role; the school timetable and school policies; features of the school smartphone policy; perceived time spent by school staff developing and implementing the school smartphone policy; knowledge and understanding of the school smartphone policy; and compliance with the school smartphone policy. The SLT member is sent the online survey by email.

#### Accelerometers

Pupils are asked to wear a wrist worn GENEActiv accelerometer watch for 24 hours a day for the subsequent 7 days after completing the survey.^64^ Watches are worn on the non-dominant wrist during all activities, including water-based activities. Accelerometers are initialised to collect data in 100 Hz. Data will be analysed in R to produce physical activity and sleep outcomes (table 3).

#### Document analysis

School policy documents and handbooks related to smartphones, social media, pupil behaviour, mental health/well-being, e-safety/IT policy and the wider school aims and ethos are collected. Documents related to the school timetable are also collected so that time spent in physical education, and time allocated for breaks and lunch can be quantified for each school.

### Case study schools

Six case study schools are being purposively sampled from the 30 participating schools in relation to two school characteristics: (1) smartphone policy type and duration to ensure a balance of restrictive (n=3) versus permissive policies (n=3), and a range of length of policy implementation in the six case studies; and (2) schools from low, medium and high area of deprivation, measured by the IDACI. An additional £300 compensation is allocated to case study schools.

Across the case study schools, up to 36 focus groups (FGs) will be completed with adolescents (n=12), school staff (n=12) and parents/carers (n=12). Data collection is taking place following the school level data collection (ie, surveys, accelerometers and document analysis) and following obtaining written consent (or assent). In each case study school four to six FGs are completed: adolescents (n=2), school staff (n=1–2) and parents/carers (n=1–2). Each FG comprises four to six members and aims to balance gender and ethnicity (where possible). For adolescents, one FG is completed with year 8 (age 12–13) and one with year 10 (age 14–15) pupils. School staff FGs include SLT, school governors, teachers and support staff (admin, caretakers, teaching assistants). Parents/carers of pupils from within the school (excluding sixth form, age 16–19) are recruited.

FGs are led by research staff employing established elicitation and semistructured interview techniques.^65 66^ Each FG lasts approximately 60 min and takes place in school or online. Data are collected from voice transcription.

### Data analysis

#### Analysis of pupil outcomes

The primary analysis will examine the association between pupil mental well-being and school policy type (restrictive/permissive). Multilevel linear models will be developed, accounting for repeated measures, clustering of classes and schools and adjusting for the school-level variables included in the propensity score estimations alongside relevant individual-level sociodemographic variables. Secondary analyses will use the same modelling approach investigating differences in the secondary (behavioural and mental health) and intermediary (smartphone and social media use) outcomes between school policy groups. Differential association between school policy and the primary, secondary and intermediary outcomes will also be explored across: (1) socioeconomic position; (2) gender; and (3) ethnicity by including relevant interaction terms in the developed models.

#### Analysis of school policies, documents and websites

School policies, documents (eg, school handbook) and relevant website content will be analysed using document analysis.^67^ This will provide an overview of smartphone policy content, rationale and communication and how schools support pupils to use their smartphones and social media, as well as their mental well-being. We will adopt a comparative approach to compare restrictive and permissive policies.

#### Economic analysis

In view of the multiple outcomes of interest, complex nature of school budgets and emergent nature of economic evaluation of school-based interventions, a cost–consequence analysis will be conducted through the relevant data collected (table 3). This approach has been previously employed for school-based interventions.^68^ In addition to the WEMWBS and CHU-9D outcomes, secondary behavioural and health outcomes outlined in the intervention logic model will be included (figure 1). An exploratory cost-utility analysis from the payer (school) perspective will be conducted to compare incremental education costs and incremental Quality-Adjusted Life-Years associated with restrictive daytime smartphone use.

#### Qualitative/case study analysis

Given the purpose of the case study method to understand complexity and situatedness,^69^ coupled with the overarching aim of this study to compare schools that have restrictive and permissive smartphone policies, it seems appropriate to analyse the case study data taking a dual approach. Thus, each case study will be analysed individually using a thematic analysis,^70^ and then multiple case analysis will be adopted.^71^ Following this approach, the findings will then be reported in detailed ‘vertical’ case reports on single schools, and in multiple case thematic analysis.^71^

### Patient and public involvement

During research plan development, a teacher member of the investigator team advised on the study. We also consulted with adolescents and teachers through an online survey (teachers n=40) and five FG consultations (teachers n=11; adolescents n=20). In addition, we undertook analysis of school websites to determine the nature of school smartphone policies. Information gathered through these activities helped to inform and refine the study design, including categorisation of school policy types, research questions, data collection methods, primary and secondary outcomes and the logic model (figure 1).

The approach to PPI during the SMART Schools Study has been constructed based on National Institute for Health Research guidelines.^72 73^ We are engaging with two groups throughout the study: (1) adolescents (from secondary schools); and (2) adults (from schools/teachers, parents/families, local/national health organisations and policy). These groups are directly impacted by the research and are representative of key stakeholders who would act on the findings. We have one PPI lead and a PPI coapplicant, who are leading on PPI activities. Throughout the study there will be four online meetings per group which will focus on: (1) designing and managing study procedures; (2) undertaking the research; and (3) dissemination. The results and conclusions from each PPI group meeting will be reported using Guidance for Reporting Involvement of Patients and the Public (GRIPP2) checklist^74^ for reporting PPI in research.

## Ethics and dissemination

### Ethical and regulatory considerations

Full ethical approval was obtained from the University of Birmingham’s Science Technology, Engineering and Mathematics Research Ethics Committee on 8 July 2022 (ERN_22-0723).

For participation in the study, parents/carers of pupil participants are not asked for active consent but are given the opportunity to complete and return a form to opt their child out of taking part in the study. Pupil, teacher, SLT and parent/carer participants are asked to provide online or written consent (or assent).

We have also developed a safeguarding protocol for conducting research on mental health with adolescents. This safeguarding protocol has been developed in the context of our measure for depression (PHQ-9) that detects whether adolescents in our sample may have had self-harm or suicidal thoughts in the past 2 weeks. The protocol outlines key procedures during recruitment, data collection and debriefing periods to safeguard pupils, and include steps related to confidentiality and establishing efficient, secure and effective communication pathways between the research team and school leaders.

Study sponsorship is provided by the University of Birmingham, with provision of research related costs supported by the National Institute for Health Research (NIHR). Data management and storage is compliant with the University of Birmingham’s policies and procedures. Participant data from online surveys and other sources will be pseudo-anonymised, stored on a University of Birmingham secure server and retained for 10 years through the University’s Research Data Archive. Data from this study will be owned by the University of Birmingham.

Study oversight will be guided by an independently chaired Study Steering Committee (SSC) and a Data Management and Ethics Committee (DMEC). A study monitoring plan has been developed and agreed on by the SSC and DMEC. The current protocol has been reviewed and agreed on by all members of the SSC and DMEC.

### Dissemination

Dissemination activities will be co-produced with our PPI participants. We have planned the following dissemination outputs: policy briefings and research summaries for agencies on the impact of school smartphone policies on adolescent mental well-being; guidelines and resources for schools on the characteristics of school smartphone and social media policy implementation that positively influence mental well-being; blogs, podcasts, videos and infographics to raise awareness and understanding of relationships between smartphones and social media and mental well-being; an NIHR public report that summarises the main project findings; and peer review and open access publications focused on the main study findings.

## Supplementary Material

Reviewer comments

Author's manuscript
