# Supplementary material for: Smartphones, social Media and Adolescent mental well-being: the impact of school policies Restricting dayTime use—protocol for a natural experimental observational study using mixed methods at secondary schools in England (SMART Schools Study)
Source: BMJ Open. 2023 Jul 5;13(7):e075832. doi: 10.1136/bmjopen-2023-075832 (PMC10335462; doi:10.1136/bmjopen-2023-075832)
Supplement: Author's manuscript [file bmjopen-2023-075832.draft_revisions.pdf]

BMJ Open is committed to open peer review. As part of this commitment we make the peer review history of every article we publish publicly available.

When an article is published we post the peer reviewers' comments and the authors' responses online. We also post the versions of the paper that were used during peer review. These are the versions that the peer review comments apply to.

The versions of the paper that follow are the versions that were submitted during the peer review process. They are not the versions of record or the final published versions. They should not be cited or distributed as the published version of this manuscript.

BMJ Open is an open access journal and the full, final, typeset and author-corrected version of record of the manuscript is available on our site with no access controls, subscription charges or pay-per-view fees (http://bmjopen.bmj.com).

If you have any questions on BMJ Open's open peer review process please email info.bmjopen@bmj.com

BMJ Open

The SMART Schools Study Protocol: Smartphones, social Media and Adolescent mental wellbeing: the impact of school policies Restricting dayTime use

Journal:	*BMJ Open*
Manuscript ID	bmjopen-2023-075832
Article Type:	Protocol
Date Submitted by the Author:	19-May-2023

Complete List of Authors:	Wood, Grace; University of Birmingham, School of Sport, Exercise and Rehabilitation Sciences; University of Birmingham Institute for Mental Health<br>Goodyear, Victoria; University of Birmingham School of Sport and Exercise Sciences; University of Birmingham Institute for Mental Health<br>Adab, Peymane; University of Birmingham Institute of Applied Health Research, Public Health<br>Al-Janabi, Hareth; University of Birmingham Institute for Mental Health; University of Birmingham Institute of Applied Health Research<br>Fenton, Sally; University of Birmingham School of Sport and Exercise Sciences, School of Sport, Exercise and Rehabilitation Sciences<br>Jones, Kirsty; Services for Education<br>Michail, Maria; University of Birmingham Institute for Mental Health; University of Birmingham School of Psychology<br>Morrison, Breanna; University of Birmingham Institute of Applied Health Research<br>Patterson, Paul; University of Birmingham Institute for Mental Health; Birmingham Women's and Children's NHS Foundation Trust<br>Sitch, Alice; University of Birmingham Institute of Applied Health Research; NIHR Birmingham Biomedical Research Centre, Institute of Translational Medicine<br>Wade, Matthew; ukactive Research Institute; Sheffield Hallam University College of Health Wellbeing and Life Sciences, Advanced Wellbeing Research Centre<br>Pallan, Miranda; University of Birmingham Institute of Applied Health Research

Keywords:	Adolescent, MENTAL HEALTH, Schools

SCHOLARONE™
Manuscripts

The SMART Schools Study Protocol: Smartphones, social Media and Adolescent mental wellbeing: the impact of school policies Restricting dayTime use

Authors: Grace Wood[1,2], Victoria A. Goodyear[1,2*], Peymane Adab[3], Hareth Al-Janabi[2,3], Sally Fenton[1], Kirsty Jones[4], Maria Michail[2,5], Breanna Morrison[3], Paul Patterson[2,6], Alice Sitch[3,7], Matthew Wade[8,9], Miranda Pallan[3].

Institutional Affiliations:
[1]School of Sport, Exercise and Rehabilitation Sciences, University of Birmingham UK
[2]Institute for Mental Health, University of Birmingham UK
[3]Institute of Applied Health Research, University of Birmingham UK
[4]Services for Education, Birmingham UK
[5]School of Psychology, University of Birmingham UK
[6]Birmingham Women's and Children's NHS Foundation Trust, Birmingham UK
[7]NIHR Birmingham Biomedical Research Centre, University Hospitals Birmingham NHS Foundation Trust and University of Birmingham, Institute of Translational Medicine, Birmingham UK
[8]ukactive, ukactive Research Institute, London UK
[9]Advanced Wellbeing Research Centre, College of Health, Wellbeing and Life Sciences, Sheffield Hallam University, Sheffield, UK

***Corresponding Author:** Dr Victoria Goodyear: v.a.goodyear@bham.ac.uk

For peer review only

For peer review only - http://bmjopen.bmj.com/site/about/guidelines.xhtml

ABSTRACT

Introduction: Smartphone and social media use is prevalent during adolescence, with high levels of use associated with lower levels of mental wellbeing. Secondary schools in the UK have introduced policies that restrict daytime use of smartphones and social media, but there is no evaluation on the impact of these policies on adolescent mental wellbeing. The SMART Schools Study aims to determine the impact of daytime restrictions of smartphone and social media use on indicators of adolescent mental wellbeing, anxiety, depression, physical activity, sleep, classroom behaviour, attainment and addictive social media use.

Methods and analysis*:* Secondary schools within a 100-mile radius of the recruiting centre in the West Midlands (UK) have been categorised into two groups: Schools that restrict (intervention) and permit (comparator) daytime use of smartphones. Employing propensity scores to guide a stratified random sampling approach, we aim to recruit 30 schools (20 restrictive, 10 permissive) and 1170 pupils aged 12-13 and 14-15 years. We will collect data on mental wellbeing, anxiety and depressive symptoms, addictive social media use, sleep and physical activity through online surveys with pupils, and accelerometers. Policy implementation measures and data on individual pupil factors will be collected through surveys with school staff. We will explore smartphone policy content, development, and implementation through document analysis of school policies and websites. Six case study schools will be selected and focus groups with school staff, parents/carers and pupils will explore individual, school and family/home factors that influence relationships between school smartphone policies, smartphone and social media use, and mental wellbeing. Economic evaluation will be completed through a cost-consequence analysis from an education sector perspective.

Ethics and dissemination: Ethical approval was obtained from the University of Birmingham's Ethics Committee (ERN_22-0723). Findings will be disseminated through policy briefings, resources for secondary schools, social media, public reports, and peer reviewed open access publications.

Trial and registration: ISRCTN77948572 and CRN 52232

Strengths and Limitations of this study

- This study addresses a knowledge gap by exploring the impact of school phone policies that restrict daytime use of smartphones and social media on adolescent mental wellbeing and associated behaviours, and the cost implications of these policies from the education sector perspective.
- Pupil outcomes will be collected from; an online survey that includes validated measures for mental wellbeing, anxiety and depressive symptoms, addictive social media use, motives for social media use, health-related quality of life and demographic data; and from accelerometers measuring physical activity and sleep.

For peer review only

- Pupils will self-report data in the online survey on time spent on their smartphone and specific social media apps by using data from their phones using iOS or Android apps.
- Data on pupil attainment, classroom behavior, implementation of school phone policies, and variables for an economic evaluation will be collected from school staff surveys and document analysis.
- Qualitative data will be collected from case study schools to further explore how individual, school and family/home factors influence relationships between school phone policies, phone/media use and mental wellbeing.

KEY WORDS

Smartphones, Social Media, Adolescent Health, Mental Health, Physical Activity, Secondary Schools, School Policy

For peer review only

INTRODUCTION

Globally, mental disorders (e.g. anxiety and depression) are the leading cause of disability in adolescents (age range 10-19) [1, 2]. In the UK, one fifth of adolescents (20%) are reported to have a mental health disorder [3], mostly anxiety and depression [2]. Half of all mental health disorders start before the age of 14 [4, 5], and if left untreated, mental health problems are highly likely to persist well into adulthood [2, 6]. Poor mental wellbeing also negatively affects other aspects of adolescents' lives, including cognitive, social and physical behaviours [6, 7]. For example, poor mental wellbeing is associated with higher rates of disruptive behaviour, school absence and lower educational attainment [8, 9]. Sleep problems are also common among adolescents diagnosed with anxiety and depression, and evidence suggests a bidirectional relationship between sleep disturbances and mental health problems [10]. Levels of physical activity also decline significantly during adolescence, and this coincides with increased onset of mental health problems [4, 11]. Hence, adolescence is a crucial period for mental health intervention [7], and there is a pressing need to improve and develop approaches to mental health prevention and intervention [6].

Smartphone and social media use is prevalent during adolescence, and accounts for the majority of their overall screen time [12, 13]. In the UK, most adolescents own a smartphone (98%), and are reported to be active users of social media (93%) [14], with comparable trends reported in other Western populations [15]. Samples in the US and the UK (2021-2022) estimate that the time adolescents spend on smartphones and social media ranges between one and a half hours and eight and a half hours per day [12-14, 16, 17], with most adolescents spending between one and three hours per day [14, 16, 17]. Problematic social media use is also prevalent, with 12% of adolescents in England reported to exhibit addictive use behaviours [18].

In moderation, smartphone and social media use (e.g., <2 hours per day) can be advantageous for mental wellbeing and mental health [19-24], as well as other associated health and behavioural outcomes (e.g. sleep, physical activity, classroom behaviour and attainment). However, at higher levels of use, the reverse effect tends to be seen, with increasing time spent on smartphones and social media associated with decreasing levels of mental wellbeing and higher levels of anxiety and depression

For peer review only - http://bmjopen.bmj.com/site/about/guidelines.xhtml

[17, 19-21, 23, 25, 26]. Poor academic performance, disruptive classroom behaviour and less time spent in physical activity and sleep are also more likely in adolescents who spend a greater proportion of time on smartphones and/or social media [19-23, 25, 26]. Reducing the time adolescents spend on smartphones and social media is thus a plausible intervention to improve mental wellbeing, possibly operating through improving the related behavioural outcomes (e.g., physical activity, sleep, academic performance, classroom behaviour). However, uncertainties in the strength of associations between smartphone/social media and mental wellbeing exist, and this is mainly due to reliance on self-reported use [15, 27-29]. Furthermore, individual (e.g. gender, age, socio-economic status) [13, 16, 30-33] and family/home (e.g. parental usage and attitudes toward technology) [34-37] factors are also likely to impact on relationships between smartphone/social media use and mental wellbeing.

There is considerable evidence that school-based interventions can have beneficial effects on adolescent mental wellbeing and associated behavioural outcomes (e.g., sleep, physical activity, classroom behaviour and attainment) [38-41]. Whole-school environment interventions that promote lifestyles conducive to good health are reported to have a more pronounced effect on mental wellbeing than individual approaches targeting knowledge and beliefs [38, 42]. A whole-school approach targets physical and social influences of health, and through the alignment of school policies, values and practices with effective school leadership, the whole school approach seeks to promote a set of values, attitudes and behaviours that encourage the development and maintenance of positive physical, social, cognitive and emotional habits [38, 40]. Evidence suggests that whole school policies related to health and wellbeing can: (i) reduce overall screen time; (ii) positively influence mental wellbeing; and (iii) improve physical activity, sleep, educational attainment and reduce disruptive classroom behaviour [43-45]. Therefore, whole school policies aiming to influence smartphone and social media use have the potential to positively impact on adolescents' mental wellbeing.

School phone policies that restrict daytime phone/media use are an example of a current whole-school environment intervention. In the UK, Australia, Sweden, Czech Republic and elsewhere, many schools have introduced school policies that restrict daytime use of smartphones in order to reduce classroom disruptive behaviour and

For peer review only - http://bmjopen.bmj.com/site/about/guidelines.xhtml

cyberbullying incidents and improve attainment [44, 46, 47]. We suggest that these policies have the potential to lower the overall time adolescents spend on the smartphones/social media, which may improve mental wellbeing and associated behavioural outcomes. However, there is currently no evaluation of the effect of school smartphone policies on mental wellbeing and there is limited evidence on how smartphone policies are implemented in schools [48].

The SMART Schools Study aims to determine the impact of school daytime restrictions of smartphone and social media use on adolescent mental wellbeing (primary outcome), anxiety, depression, physical activity, sleep, classroom behaviour, attainment, and addictive social media use. We will do this by comparing impacts in two different secondary school phone policy contexts: (i) schools that do not permit smartphone use during recreational time in the school day (intervention); and (ii) schools that permit smartphone use during recreational time (breaks/lunchtimes) (comparator). We will also explore how variation in school-based, individual and family/home factors influence the relationship between school phone policies, smartphone and social media use and mental wellbeing. We will conduct an economic evaluation in the form of a cost-consequence analysis from an education sector perspective.

METHODS AND ANALYSIS

This is a natural experimental observational study using mixed methods. Quantitative and qualitative data will be collected from all schools in the sample (n = 30) to compare outcomes between restrictive (intervention) and permissive (comparator) school policies and to complete an economic evaluation. Qualitative data will be collected from six case study schools to understand the contextual factors that could influence relationships between school policies, smartphone and social media use and mental wellbeing.

Intervention: School Smartphone Policies that Restrict Daytime Use

The intervention and data capture are directed by our logic model (Figure 1) which integrates multiple theories and evidence. First, we adhere to displacement theories

For peer review only - http://bmjopen.bmj.com/site/about/guidelines.xhtml

[19] to propose that reducing the time adolescents spend on phones/media (i.e. restricting school time use) is optimal for mental wellbeing. Overuse can displace other mental wellbeing promoting activities (e.g. sleep and physical activity) and very low use can deprive adolescents of interactions that support mental wellbeing (e.g., affect and relationships) [19, 21, 23]. Second, psychological motives drive phone/media use; motives related to enhancement and social interactions promote mental wellbeing, and motives related to coping and conformity (e.g. Fear of Missing Out) are associated with problematic use (addiction) and poor mental wellbeing [49, 50]. Hence the school policy and ethos have the potential to influence adolescents' motives for using phones/media, which may impact on mental wellbeing. Third, the ecological model of social influence proposes three agents that shape wellbeing and technology use, including the school environment, home/family and individual factors [51]. Finally, policy enactment and implementation process models [39, 52, 53] identify that school policy implementation effects will be shaped by social processes (e.g., training, leadership, compliance, administration, family-school interactions).

[Figure 1]

Figure 1. Logic model and theory of change for the influence of school policies that restrict daytime use of smartphones on mental wellbeing and other associated behavioural and health outcomes in adolescents.

Informed by our Patient and Public Involvement (PPI) activities and school smartphone policy analysis, the components of the intervention (i.e., restrictive school smartphone policies) are presented in Table 1. In Table 2 we have outlined variations in school smartphone policies and our classification of these variations into two school policy groups: restrictive (intervention) and permissive (comparator).

For peer review only - http://bmjopen.bmj.com/site/about/guidelines.xhtml

Table 1. Components of the intervention, guided by the template for intervention description and replication (TIDieR) checklist [54].

TiDieR Components	SMART Schools Intervention
Description	School policy prohibiting the use of smartphones during the school day
Materials	The policy may be communicated to parents/carers and adolescents in a variety of ways, such as through school information packs, assemblies, letters and/or the school website
Procedures	Adolescents are not permitted to use their smartphones during lessons or recreational time in the school day, and their smartphones must not be seen in school during these times
Provider	Schools (or Multi-Academy Trusts*) develop their own policies, often in consultation with school staff, parents and/or school governors, and in relation to the school ethos
Mode of Delivery	The Senior Leadership team (SLT) and school staff enact the school policy and are required to administer behavioral consequences for adolescents who use their smartphone during the day, such as smartphone confiscation, detention and/or parent-school meeting
Time Period	Schools vary in terms of how long their school smartphone has been implemented
Tailoring	Schools have developed policies according to their specific school contexts (or Multi-Academy Trusts*). Policies usually apply to the whole school, although in some schools 6th form pupils (age 16+) may be permitted to use their smartphones during the school day (this age group will not be investigated in this study)
Adherence and Fidelity	The degree to which pupils and teachers adhere to the school policy, and parents/carers are in support of the policy varies across schools

* Multi-Academy Trusts (MAT) –non-profit companies that manage more than one academy [55].

For peer review only

Table 2. Classifications of Variations in School Smartphone Policies as Restrictive (Intervention) and Permissive (Comparator).

Restrictive School Smartphone Policies (Intervention)	Permissive School Smartphone Policies (Comparator)
Allow smartphones onto school premises but insist these are not to be used during the school day and are turned off and out of sight	Allow pupils to carry smartphones and use them at any time point during the day
Allow smartphones onto school premises, but only allow use if sanctioned by teaching staff for educational activities (e.g., use of calculator)	Allow pupils to carry smartphones and use them at specific time points during the day (e.g., breaks and lunch)
Allow smartphones onto premises but insist they are left in a specified place during the school day e.g., school reception or lockers	Allow pupils to carry smartphones and use them for personal use with consent from school staff
Pupils are not allowed to carry their smartphones onto school premises at any time	Allow pupils to carry smartphones and use within designated areas or zones

Study Setting

The sampling frame comprises UK secondary schools (ages 11-19) located within a 100-mile radius of the recruiting centre in the West Midlands. 64 local authorities are included in the sampling frame from the West Midlands, East Midlands, East, South East, South West and North West. The schools are situated in regions of high and low levels of deprivation and in areas that have high [56] and low proportions of Black, Asian and Minority Ethnic (BAME) groups [57, 58]. Schools other than state-funded mainstream schools (special schools, pupil referral units and independent schools) were excluded because it was expected that there would be additional influences on mental wellbeing. Schools that did not have an accessible smartphone policy and/or had different smartphone policies for the year groups 8 and 10, and schools with missing data that was required for the propensity score estimation (see sampling and participants) were also excluded (n = 10,810). A total of 1345 secondary schools are included in our sampling frame; 1220 schools with policies classified as restrictive (intervention) and 125 as permissive (comparator).

For peer review only - http://bmjopen.bmj.com/site/about/guidelines.xhtml

For peer review only

Sampling and participants

To improve the comparability of the two school groups, stratified sampling based on propensity scores was employed [59]. We obtained routine data from the Department for Education on the following school characteristics: region; school type; urban or rural; total pupil roll size; Income Deprivation Affecting Children Index (IDACI) [60]; inclusion of a sixth form; selective or non-selective admissions policy; religious affiliation; and the proportion of pupils with the following characteristics: male; from BAME groups; English as an Additional Language; eligible for free school meals; and Special Education Needs. Propensity scores were calculated using restrictive or permissive school smartphone policies as the outcome and school characteristics as explanatory variables. Propensity score terciles were then used to create three groups with subsequent division by restrictive or permissive policy type, resulting in 6 distinct sampling groups. Schools in each group have been randomly ordered and are being invited sequentially to participate, aiming to recruit six to seven schools from each restrictive tercile and three to four from each permissive tercile to achieve a sample size of 20 schools with restrictive and 10 schools with permissive policies.

In participating schools, two classes of mixed ability year 8 (age 12-13) and year 10 (age 14-15) pupils are recruited. We are focusing on adolescents in this age range because of the age of onset and prevalence of mental illness [5], and the prevalence of smartphone and social media use by adolescents aged 12-15 [7, 14, 61]. Within this age range we may also be able to observe potential differences in smartphone/ social media use relationships with wellbeing [33]. For example, year 8 pupils are likely to be newer users of phones/media and physical activity levels begin to decline at this age, particularly among girls [7, 13]. In year 10, pupils are more likely to be established phone/media users, mental wellbeing tends to be lower and this age group are approaching the peak onset of mental health conditions [4]. In addition to adolescent participants, the form teacher for each class recruited (or an equivalent teacher responsible for the class) and a member of SLT who is responsible for the school smartphone policy are recruited from each school.

Sample size calculation

For peer review only - http://bmjopen.bmj.com/site/about/guidelines.xhtml

To account for the imbalance of schools in our sample that have permissive (n= 125) and restrictive policies (n =1220), we are recruiting schools using a 2:1 (restrictive: permissive) ratio. The primary outcome of mental wellbeing will be measured using the Warwick-Edinburgh Mental Well-Being Scale (WEMWBS; score range =14-70) [62]. To detect a mean difference in score of three points (considered the minimum clinically important difference [62] between the 2 school groups), assuming a SD of 6.8 [63] and an ICC of 0.1 (a conservative estimate [64]), with 90% power and 5% significance, we require 20 schools in the restrictive and 10 schools in the permissive smartphone policy groups, with an average cluster size of 39 (1170 pupil participants in total; 780 in the restrictive, and 390 in the permissive policy groups).

In each participating class, we aim to recruit a minimum of 19-20 pupils (67% if estimated class size n = 30). In studies with multiple layers of clustering (here classes within schools), it is conservative to treat clusters within clusters as one larger cluster, which is the approach used here [65].

Recruitment

School recruitment commenced in September 2022 and will continue until December 2023. A study advertisement has been emailed to all schools in the sampling frame. Following our propensity sampling approach, schools are then invited by post and email with a telephone follow-up. In participating schools, a School Liaison Member of staff (SLM) is identified and a school-university contract outlining expected commitments signed. Subsequently, a member of SLT responsible for the smartphone policy, and pupils and teachers from the year 8 and year 10 classes are recruited. £600 compensation is allocated to each school and a £5 voucher per pupil participant.

Parents/carers are provided with written detailed information about the study, what their child's participation will involve and how their child's data will be processed. Schools are asked to assist in the distribution of this information to parents/carers in different formats (e.g., email, post, text messages, website etc.). Parents/carers are asked for active consent but are given the opportunity to complete and return a form to opt their child out of the study. Pupil, teacher and SLT participants receive written

For peer review only

information about the study and are asked to complete either an online or written consent (or assent) form.

Data collection

Data collection methods include self-administered surveys for pupils, teachers and a member of the SLT; accelerometer measured physical activity and sleep for pupils; and document analysis. All online surveys are completed using university-approved online survey software (REDCap). Table 3 provides an overview of outcomes, measures and timepoints of evaluation.

Table 3. Overview of Outcomes, Measures and Timepoints of Evaluation.

Outcome	Outcome Measures	Timepoint(s) of evaluation
**Primary Outcome**		
Mental Wellbeing	Warwick-Edinburgh Mental Well-Being Scale (WEMWBS) [62] / pupil survey	Measured over the past 2 weeks at 2 time points, 4-8 weeks apart
**Secondary Outcomes**		
Anxiety Symptoms	Generalized Anxiety Disorder Assessment (GAD-7) [66] / pupil survey	1 time point measured over the past 2 weeks
Depressive Symptoms	Patient Health Questionnaire (PHQ-9) [67] / pupil survey	1 time point measured over the past 2 weeks
Addictive Use	Problematic Social Media use Scale [49, 50] / pupil survey	1 time point based on usual use
Sleep	Sleep quality (indicated by sleep duration and sleep efficiency / accelerometers<br><br>Time pupils go to sleep and wake time on a school day and a weekend day / pupil survey	Over 7 days / usual behaviours
Physical Activity	Physical activity (total and moderate-to-vigorous intensity (MVPA) / accelerometers<br><br>Active travel and sports/activity club engagement/ pupil survey	Over 7 days / usual behaviours<br><br>Usual behaviours

For peer review only - http://bmjopen.bmj.com/site/about/guidelines.xhtml

Attainment	Assessment of whether pupils are below, above or working at their target grade in English and Maths / teacher survey	1 time point – most recent assessment
Disruptive Classroom Behaviour	Pupil Behaviour Questionnaire [68] / teacher survey	1 time point – current assessment
**Intermediate Outcomes**		
Smartphone Use Duration	3 measures: (i) within school; (ii) over 24-hour period on a school day; (iii) on a weekend day / data captured from iOS and Android smartphones and will be self-reported through the pupil survey	1 time point
Social Media Use Duration	3 measures: (i) within school; (ii) over 24-hour period on a school day; (iii) on a weekend day / data captured from iOS and Android smartphones and will be self-reported through the pupil survey	1 time point
Motives for Social Media Use	Social Media Motives Questionnaire / pupil survey	1 time point measured over past 12 months
**Policy Implementation Measures**		
Intervention Components (School Level Data): School Timetable; School Policies (smartphone, mental health, behaviour, e-safety): School Smartphone Policy Details (e.g., rules/key features; time period of implementation; policy communication and understanding; adherence and fidelity and policy rationale)	School Policy Documents<br>School Handbooks<br>School Website Content<br>SLT survey<br>Teacher survey<br>Pupil survey	1 time point
Individual Factors (Pupils): age, gender, ethnicity, socio-economic position, eligibility for Free School Meals, and whether pupils have English as an Additional Language and/or Special Educational Needs	Pupil Survey<br>Teacher Survey	1time point
Contextual Factors: School, Individual, Family/Home	Focus Groups with school staff, pupils and parents/carers of school pupils	1 time point
**Economic Variables**		
Teacher Time Spent Managing Policy	SLT Survey<br>Teacher survey	1 time point

For peer review only

Indirect Time Spent Managing Behavioural Problems<br>Other Related Costs		
Health-related quality of life / Quality Adjusted Life Years	The Child Health Utility Instrument (CHU9D) [69] / pupil survey	1 time point / current assessment

Pupil Online Survey

Pupils complete an online survey at one time point (in school time) that includes validated measures for mental wellbeing, anxiety and depressive symptoms, addictive social media use, motives for social media use, health-related quality of life and demographic variables (e.g., age, gender) (Table 3). Within the survey pupils are asked to self-report data on their physical activity levels, sleep and phone/media use, and by using data from their iOS or Android apps, they additionally self-report data on time spent on their smartphone and social media apps (Table 3). Within the survey pupils are also asked to report on their knowledge and understanding of the school smartphone policy and compliance with the school smartphone policy The survey is completed on encrypted tablets, using a portable Wi-Fi hub owned by the research team.

Secondary measure of mental wellbeing

A second online survey (in school time) to measure mental wellbeing (Table 3) is completed four to eight weeks after pupils have completed the initial online survey. The SLM is sent an email link for pupils to complete.

Teacher Online Survey

Data are collected from the form teacher (or an equivalent teacher responsible for the class) for each participating pupil on: pupil attainment; classroom behavior; and whether pupils are eligible for free school meals, have a special educational need, or have English as an additional language (Table 3). Within the survey teachers are also asked to report on their knowledge and understanding of the school smartphone policy, compliance with the school smartphone policy and to report on the time they

For peer review only - http://bmjopen.bmj.com/site/about/guidelines.xhtml

spend implementing the school smartphone policy (Table 3). Teachers are sent the online survey by email.

Senior Leadership Team Online Survey

The SLT member responsible for the school smartphone policy is asked to complete survey questions on: the SLT member's role; the school timetable and school policies; features of the school smartphone policy; perceived time spent by school staff developing and implementing the school smartphone policy; knowledge and understanding of the school smartphone policy; and compliance with the school smartphone policy. The SLT member is sent the online survey by email.

Accelerometers

Pupils are asked to wear a wrist worn GENEActiv accelerometer watch for 24 hours a day for the subsequent seven days after completing the survey [70]. Watches are worn on the non-dominant wrist during all activities, including water-based activities. Accelerometers are initialised to collect data in 100Hz. Data will be analysed in R to produce physical activity and sleep outcomes (Table 3).

Document Analysis

School policy documents and handbooks related to smartphones, social media, pupil behavior, mental health/wellbeing, e-safety/IT policy and the wider school aims and ethos are collected. Documents related to the school timetable are also collected so that time spent in physical education, and time allocated for breaks and lunch can be quantified for each school.

Case Study Schools

Six case study schools are being purposively sampled from the 30 participating schools in relation to two school characteristics: (i) smartphone policy type and duration to ensure a balance of restrictive (n=3) vs permissive policies (n=3), and a range of length of policy implementation in the six case studies; and (ii) schools from

For peer review only

low, medium and high area of deprivation, measured by the Income Deprivation Affecting Children Index (IDACI). An additional £300 compensation is allocated to case study schools.

Across the case study schools, up to 36 focus groups (FG) will be completed with adolescents (n=12), school staff (n=12) and parents/carers (n=12). Data collection is taking place following the school level data collection (i.e., surveys, accelerometers and document analysis) and following obtaining written consent (or assent). In each case study school four to six FGs are completed: adolescents (n=2), school staff (n=1-2) and parents/carers (n=1-2). Each FG comprises four to six members and aims to balance gender and ethnicity (where possible). For adolescents, one FG is completed with year 8 (age 12-13) and one with year 10 (age 14-15) pupils. School staff FGs include SLT, school governors, teachers and support staff (admin, caretakers, teaching assistants). Parents/carers of pupils from within the school (excluding 6th form, age 16-19) are recruited.

FGs are led by research staff employing established elicitation and semi-structured interview techniques [71, 72]. Each FG lasts approximately 60 minutes and takes place in school or online. Data are collected from voice transcription.

Data Analysis

Analysis of pupil outcomes

The primary analysis will examine the association between pupil mental wellbeing and school policy type (restrictive/permissive). Multilevel linear models will be developed, accounting for repeated measures, clustering of classes and schools and adjusting for the school-level variables included in the propensity score estimations alongside relevant individual-level sociodemographic variables. Secondary analyses will use the same modelling approach investigating differences in the secondary (behavioural and mental health) and intermediary (smartphone and social media use) outcomes between school policy groups. Differential association between school policy and the primary, secondary and intermediary outcomes will also be explored across: (i) socioeconomic position; (ii) gender; and (iii) ethnicity by including relevant interaction

For peer review only - http://bmjopen.bmj.com/site/about/guidelines.xhtml

terms in the developed models.

Analysis of school policies, documents and websites

School policies, documents (e.g. school handbook) and relevant website content will be analysed using document analysis [73]. This will provide an overview of smartphone policy content, rationale and communication, and how schools support pupils to use their smartphones and social media, as well as their mental wellbeing. We will adopt a comparative approach to compare restrictive and permissive policies.

Economic analysis

In view of the multiple outcomes of interest, complex nature of school budgets and emergent nature of economic evaluation of school-based interventions, a cost-consequence analysis will be conducted through the relevant data collected (Table 3). This approach has been previously employed for school-based interventions [74]. In addition to the WEMWBS and CHU-9D outcomes, secondary behavioural and health outcomes outlined in the intervention logic model will be included (Figure 1). An exploratory cost-utility analysis from the payer (school) perspective will be conducted to compare incremental education costs and incremental Quality-Adjusted Life-Years associated with restrictive daytime smartphone use.

Qualitative/Case Study Analysis

Given the purpose of the case study method to understand complexity and situatedness [75], coupled with the overarching aim of this study to compare schools that have restrictive and permissive smartphone policies, it seems appropriate to analyse the case study data taking a dual approach. Thus, each case study will be analysed individually using a thematic analysis [76], and then multiple case analysis will be adopted [77]. Following this approach, the findings will then be reported in detailed ‘vertical’ case reports on single schools, and in multiple case thematic analysis [77].

Patient and Public Involvement (PPI)

For peer review only

During research plan development, a teacher member of the investigator team advised on the study. We also consulted with adolescents and teachers through an online survey (teachers n= 40) and five FG consultations (teachers n=11; adolescents n=20). In addition, we undertook analysis of school websites to determine the nature of school smartphone policies. Information gathered through these activities helped to inform and refine the study design, including categorization of school policy types, research questions, data collection methods, primary and secondary outcomes and the logic model (Figure 1).

The approach to PPI during the SMART Schools Study has been constructed based on National Institute for Health Research guidelines [78, 79]. We are engaging with two groups throughout the study: (i) adolescents (from secondary schools); and (ii) adults (from schools/teachers, parents/families, local/national health organizations and policy). These groups are directly impacted by the research and are representative of key stakeholders who would act on the findings. We have one PPI Lead and a PPI Co-applicant, who are leading on PPI activities. Throughout the study there will be four online meetings per group which will focus on: (i) designing and managing study procedures; (ii) undertaking the research; and (iii) dissemination. The results and conclusions from each PPI group meeting will be reported using Guidance for Reporting Involvement of Patients and the Public (GRIPP2) checklist [80] for reporting PPI in research.

ETHICS AND DISSEMINATION

Ethical and Regulatory Considerations

Full ethical approval was obtained from the University of Birmingham's Science Technology, Engineering and Mathematics Research Ethics Committee on 8th July 2022 (ERN_22-0723). We have also developed a safeguarding protocol for conducting research on mental health with adolescents. This safeguarding protocol has been developed in the context of our measure for depression (PHQ-9) that detects whether adolescents in our sample may have had self-harm of suicidal thoughts in the past two weeks. The protocol outlines key procedures during recruitment, data collection and de-briefing periods to safeguard pupils, and include steps related to confidentiality and

For peer review only

establishing efficient, secure and effective communication pathways between the research team and school leaders.

Study sponsorship is provided by the University of Birmingham, with provision of research related costs supported by the National Institute for Health Research (NIHR). Data management and storage is compliant with the University of Birmingham's policies and procedures. Participant data from online surveys and other sources will be pseudo-anonymised, stored on a University of Birmingham secure server and retained for 10 years through the University's Research Data Archive. Data from this study will be owned by the University of Birmingham.

Study oversight will be guided by an independently chaired Study Steering Committee (SSC) and a Data Management and Ethics Committee (DMEC). A study monitoring plan has been developed and agreed upon by the SSC and DMEC. The current protocol has been reviewed and agreed upon by all members of the SSC and DMEC.

Dissemination

Dissemination activities will be co-produced with our PPI participants. We have planned the following dissemination outputs: Policy briefings and research summaries for agencies on the impact of school smartphone policies on adolescent mental wellbeing; Guidelines and resources for schools on the characteristics of school smartphone and social media policy implementation that positively influence mental wellbeing; Blogs, podcasts, videos and infographics to raise awareness and understanding of relationships between smartphones and social media and mental wellbeing; an NIHR public report that summarises the main project findings; and peer review and open access publications focused on the main study findings.

For peer review only

REFERENCES

1. World Health Organization. Adolescent Health [online] 2022. https://www.who.int/health-topics/adolescent-health#tab=tab_1 (Accessed 23rd February 2022).
2. World Health Organization. Health for the World's Adolescents - A Second Change in the Second Decade. Geneva, Switzerland; 2014.
3. NHS Digital. Mental Health of Children and Young People in England 2022- wave 3 follow up to the 2017 survey [online] 2022. https://digital.nhs.uk/data-and-information/publications/statistical/mental-health-of-children-and-young-people-in-england/2022-follow-up-to-the-2017-survey# (Accessed 23rd February 2022).
4. Kessler RC, Amminger GP, Aduilar-Gaxiola S, et al. Age of onset of mental disorders: a review of recent literature. Curr Opin Psychiatry 2007; 20(4):359-64.
5. Kessler RC, Berglund P, Demler O, et al. Lifetime prevalence and age-of-onset distributions of DSM-IV disorders in the National Comorbidity Survey Replication. Arch Gen Psychiatry 2005; 62(6):593-602.
6. Colizzi M, Lasalvia A, Ruggeri M. Prevention and early intervention in youth mental health: is it time for a multidisciplinary and trans-diagnostic model for care? IJMHS 2020;14(1):23.
7. Patton GC, Sawyer SM, Santelli JS, et al. Our future: a *Lancet* commission on adolescent health and wellbeing. The Lancet 2016;387(10036):2423-78.
8. Finning K, Ukoumunne OC, Ford T, et al. The association between child and adolescent depression and poor attendance at school: A systematic review and meta-analysis 2019; 245:928-38.
9. Veldman K, Bültmann U, Stewart RE, et al. Mental health problems and educational attainment in adolescence: 9-year follow-up of the TRAILS study. PLoS One 2014;9(7):e101751.
10. Orchard F, Gregory AM, Gradisar M, et al. Self-reported sleep patterns and quality amongst adolescents: cross-sectional and prospective associations with anxiety and depression. J Child Psychol Psychiatry 2020;61(10):1126-37.
11. Zimmermann-Sloutskis D, Wanner M, Zimmermann E, et al. Physical activity levels and determinants of change in young adults: a longitudinal panel study. International Journal of Behavioral Nutrition and Physical Activity 2010;7(1):2.

For peer review only - http://bmjopen.bmj.com/site/about/guidelines.xhtml

12. Rideout V, Peebles A, Mann S, et al. Common Sense census: Media use by tweens and teens, 2021. San Francisco, CA; 2022.
13. Ofcom. Children and parents: media use and attitudes report 2022 [online]. 2022. https://www.ofcom.org.uk/__data/assets/pdf_file/0024/234609/childrens-media-use-and-attitudes-report-2022.pdf (Accessed 23rd February 2022).
14. Ofcom. Children and Parents: Media Use and Attitudes 2023 [online] 2023. https://www.ofcom.org.uk/__data/assets/pdf_file/0027/255852/childrens-media-use-and-attitudes-report-2023.pdf (Accessed 31st March 2023).
15. Orben A. Teenagers, screens and social media: a narrative review of reviews and key studies. Social Psychiatry and Psychiatric Epidemiology. 2020;55(4):407-14.
16. Scott H, Biello SM, Woods HC. Social media use and adolescent sleep patterns: cross-sectional findings from the UK millennium cohort study. BMJ Open 2019;9(9):e031161.
17. Plackett R, Sheringham J, Dykxhoorn J. The Longitudinal Impact of Social Media Use on UK Adolescents' Mental Health: Longitudinal Observational Study. J Med Internet Res 2023;25:e43213.
18. Brooks F KE, Chester K, Magnusson J, et al. Health Behaviour in School-aged Children (HSBC): World Health Organization Collaborative Cross National Study. Findings from the 2018 HSBC study for England. Hatfield, England; 2020.
19. Przybylski AK, Weinstein N. A Large-Scale Test of the Goldilocks Hypothesis. Psychol Sci 2017;28(2):204-15.
20. Przybylski AK, Orben A, Weinstein N. How Much Is Too Much? Examining the Relationship Between Digital Screen Engagement and Psychosocial Functioning in a Confirmatory Cohort Study. J Am Acad Child Adolesc Psychiatry 2020;59(9):1080-8.
21. Viner RM, Gireesh A, Stiglic N, et al. Roles of cyberbullying, sleep, and physical activity in mediating the effects of social media use on mental health and wellbeing among young people in England: a secondary analysis of longitudinal data. Lancet Child Adolesc Health 2019;3(10):685-96.
22. Yan H, Zhang R, Oniffrey TM, et al. Associations among Screen Time and Unhealthy Behaviors, Academic Performance, and Well-Being in Chinese Adolescents. Int J Environ Res Public Health 2017;14(6).
23. Royal College of Psychiatrists. Technology use and the mental health of children and young people [online] 2020. https://www.rcpsych.ac.uk/docs/default-source/improving-care/better-mh-policy/college-reports/college-report-cr225.pdf (Accessed 23rd February 2022).
24. Dienlin T, Johannes N. The impact of digital technology use on adolescent well-being. Dialogues in Clinical Neuroscience 2020;22(2):135-42.
25. Kelly Y ZA, Booker C, Sacker A. Social Media Use and Adolescent Mental Health: Findings From the UK Millennium Cohort Study. eClinicalMedicine 2019;6:59-68.
26. Twenge JM, Martin GN, Campbell WK. Decreases in psychological well-being among American adolescents after 2012 and links to screen time during the rise of smartphone technology. Emotion 2018;18(6):765-80.
27. Orben A, Przybylski AK. Screens, Teens, and Psychological Well-Being: Evidence From Three Time-Use-Diary Studies. Association for Psychological Science 2019;30:682–96.
28. Kandola A, Owen N, Dunstan DW, et al. Prospective relationships of adolescents' screen-based sedentary behaviour with depressive symptoms: the Millennium Cohort Study. Psych Med 2022;52(15):3531-9.

For peer review only

29. Sewall CJR, Bear TM, Merranko J, et al. How psychosocial well-being and usage amount predict inaccuracies in retrospective estimates of digital technology use. Mobile Media & Communication 2020;8(3):379-99.
30. Patalay P, Fitzsimons E. Development and predictors of mental ill-health and wellbeing from childhood to adolescence. Soc Psychiatry Psychiatr Epidemiol 2018;53(12):1311-23.
31. Sport England. Active Lives Children and Young People Survey: Academic Year 2021-22 [online] 2022. https://sportengland-production-files.s3.eu-west-2.amazonaws.com/s3fs-public/2022-12/Active%20Lives%20Children%20and%20Young%20People%20Survey%20Academic%20Year%202021-22%20Report.pdf?VersionId=R5_hmJHw5M4yKFsewm2vGDMRGHWW7q3E (Accessed 23rd February 2022).
32. NHS. Mental Health of Children and Yougn People in England, 2017 [PAS] [online] 2018. https://digital.nhs.uk/data-and-information/publications/statistical/mental-health-of-children-and-young-people-in-england/2017/2017 (Accessed 23rd February 2022).
33. Orben A, Przybylski AK, Blakemore S-J, et al. Windows of developmental sensitivity to social media. Nature Communications 2022;13(1):1649.
34. Roffery S. Positive Relationships. London: Springer; 2012.
35. Salway RE, Emm-Collison L, Sebire S, et al. Associations between socioeconomic position and changes in children's screen-viewing between ages 6 and 9: a longitudinal study. BMJ Open 2019;9(12):e027481.
36. Thompson JL, Sebire SJ, Kesten JM, et al. How parents perceive screen viewing in their 5–6 year old child within the context of their own screen viewing time: a mixed-methods study. BMC Public Health 2017;17(1):471.
37. Solomon-Moore E, Matthews J, Reid T, et al. Examining the challenges posed to parents by the contemporary screen environments of children: a qualitative investigation. BMC Pediatrics 2018;18(1):129.
38. Bonell C, Jamal F, Harden A, et al. Public Health Research. Systematic review of the effects of schools and school environment interventions on health: evidence mapping and synthesis. Southamptom: NIHR Journals Library; 2013.
39. Paulus FW, Ohmann S, Popow C. Practitioner Review: School-based interventions in child mental health. JCPP 2016;57(12):1337-59.
40. Glazzard J. A whole-school approach to supporting children and young people's mental health. JPMH 2019;18(4):256-65.
41. Barker R, Hartwell G, Egan M, et al. The importance of school culture in supporting student mental health in secondary schools. Insights from a qualitative study. British Educational Research Journa 2023;00:1-23.
42. Leatherdale ST, Faulkner G, Arbour-Nicitopoulos K. School and student characteristics associated with screen-time sedentary behavior among students in grades 5-8, Ontario, Canada, 2007-2008. Prev Chronic Dis 2010;7(6):A128.
43. Katapally TR, Laxer RE, Qian W, et al. Do school physical activity policies and programs have a role in decreasing multiple screen time behaviours among youth? Prev Med 2018;110:106-13.
44. Kvardova N VN. Do mobile phone bans work? Rules and the use of technology in school in relation to adolescents' behaviour. Research Report from primary and secondary schools. Brno: Masaruk University; 2019.

For peer review only

45. Dix KL, Slee PT, Lawson MJ, et al. Implementation quality of whole-school mental health promotion and students' academic performance. Child Adolesc Ment Health 2012;17(1):45-51.
46. Selwyn N, Aagaard J. Banning mobile phones from classrooms—An opportunity to advance understandings of technology addiction, distraction and cyberbullying. British Journal of Educational Technology 2021;52(1):8-19.
47. Beland L-P, Murphy R. Ill Communication: Technology, distraction & student performance. Labour Economics 2016;41:61-76.
48. House of Commons. Impact of social media and screen-use on young people's health. London: House of Commons; 2019.
49. Marino C, Vieno A, Altoè G, et al. Factorial validity of the Problematic Facebook Use Scale for adolescents and young adults. J Behav Addict. 2017;6(1):5-10.
50. Marino C, Mazzieri E, Caselli G, et al. Motives to use Facebook and problematic Facebook use in adolescents. J Behav Addict 2018;7(2):276-83.
51. Livingstone S, Sefton-Green J. The Class: Living and Learning in the Digital Age. New York: New York University Press; 2016.
52. Ball S. Education Debate. London: Policy Press; 2017.
53. Ball SJ, Maguire M, Braun A. How schools do policy. London: Routledge; 2012.
54. Hoffmann TC, Glasziou PP, Boutron I, et al. Better reporting of interventions: template for intervention description and replication (TIDieR) checklist and guide. BMJ 2014;348:g1687.
55. Haves E. Education: multi academy trusts [online] 2022. https://lordslibrary.parliament.uk/education-multi-academy-trusts/ (accessed 31st March 2022).
56. Office for National Statistics. Mapping income deprivation at a local authority level [online] 2021. https://www.ons.gov.uk/peoplepopulationandcommunity/personalandhouseholdfinances/incomeandwealth/datasets/mappingincomedeprivationatalocalauthoritylevel (Accessed 23rd February 2022).
57. Office for National Statistics. Ethnicity and National Identity 2012 [online] 2012. https://www.ons.gov.uk/peoplepopulationandcommunity/culturalidentity/ethnicity/articles/ethnicityandnationalidentityinenglandandwales/2012-12-11#:~:text=The%20West%20Midlands%20had%20a,British%20at%2079.2%20per%20cent (Accessed 23rd February 2022).
58. Office for National Statistics. Population Characteristics Research Tables 2019 [online] 2019. https://www.ons.gov.uk/peoplepopulationandcommunity/populationandmigration/populationestimates/datasets/populationcharacteristicsresearchtables (Accessed 23rd February 2022).
59. Rubin DB. Estimating causal effects from large data sets using propensity scores. Ann Intern Med 1997;127(8 Pt 2):757-63.
60. Ministry of Housing, Communities & Local Government. The English Indices of deprivation 2019 (IoD2019). London: Ministry of Housing, Communities & Local Government; 2019.
61. Ofcom. Children and parents: Media use and attitudes report [online] 2019. https://www.ofcom.org.uk/__data/assets/pdf_file/0023/190616/children-media-use-attitudes-2019-report.pdf (Accessed 23rd February 2022).
62. Warwick Medical School. Collect, score, analuse and interpret WEMWBS [online] 2020. https://warwick.ac.uk/fac/sci/med/research/platform/wemwbs/using/howto/ (Accessed 23rd February 2022).

For peer review only

63. Clarke A, Friede T, Putz R, et al. Warwick-Edinburgh Mental Well-being Scale (WEMWBS): Validated for teenage school students in England and Scotland. A mixed methods assessment. BMC Public Health 2011;11(1):487.
64. Hale DR, Patalay P, Fitzgerald-Yau N, et al. School-level variation in health outcomes in adolescence: analysis of three longitudinal studies in England. Prev Sci 2014;15(4):600-10.
65. Hemming K, Lilford R, Girling AJ. Stepped-wedge cluster randomised controlled trials: a generic framework including parallel and multiple-level designs. Stat Med 2015;34(2):181-96.
66. Löwe B, Decker O, Müller S, et al. Validation and standardization of the Generalized Anxiety Disorder Screener (GAD-7) in the general population. Med Care 2008;46(3):266-74.
67. Kroenke K, Spitzer RL, Williams JB. The PHQ-9: validity of a brief depression severity measure. J Gen Intern Med 2001;16(9):606-13.
68. Allwood M, Allen K, Price A, et al. The reliability and validity of the pupil behaviour questionnaire: a child classroom behaviour assessment tool. Emotional and Behavioural Difficulties 2018;23(4):361-71.
69. Furber G, Segal L. The validity of the Child Health Utility instrument (CHU9D) as a routine outcome measure for use in child and adolescent mental health services. Health and Quality of Life Outcomes 2015;13(1):22.
70. Dillon CB, Fitzgerald AP, Kearney PM, et al. Number of Days Required to Estimate Habitual Activity Using Wrist-Worn GENEActiv Accelerometer: A Cross-Sectional Study. PLoS One 2016;11(5):e0109913.
71. Flick U. The SAGE Handbook of Qualitative Data Collection [online] 2018. https://sk.sagepub.com/reference/the-sage-handbook-of-qualitative-data-collection (Accessed 31st March 2023).
72. Galletta A. Mastering the Semi-Structured Interview and Beyond: From Research Design to Analysis and Publication. New York: New York University Press; 2013.
73. Dalglish SL, Khalid H, McMahon SA. Document analysis in health policy research: the READ approach. Health Policy and Planning 2020;35(10):1424-31.
74. Charles JM, Harrington DM, Davies MJ, et al. Micro-costing and a cost-consequence analysis of the 'Girls Active' programme: A cluster randomised controlled trial. PLoS One 2019;14(8):e0221276.
75. Stake RE. The art of case study research. Thousand Oaks, California: SAGE; 1995.
76. Braun V, Clarke V. Using thematic analysis in psychology. Qualitative Research in Psychology 2006;3(2):77-101.
77. Stake RE. Multiple case study analysis. New York: Guilford Press; 2006.
78. NIHR. Briefing notes for researchers- public involvement in NHS, health and social care research: NIHR [online] 2021. https://www.nihr.ac.uk/documents/briefing-notes-for-researchers-public-involvement-in-nhs-health-and-social-care-research/27371#who-should-i-involve (Accessed 23rd February 2022).
79. NIHR. Different experiences: A framework for consdiering who might be involved in research: NIHR [online] 2021. https://www.nihr.ac.uk/documents/different-experiences-a-framework-for-considering-who-might-be-involved-in-research/27387 (Accessed 23rd February 2022).
80. Staniszewska S, Brett J, Simera I, et al. GRIPP2 reporting checklists: tools to improve reporting of patient and public involvement in research. BMJ 2017;358:j3453.

For peer review only

Author contributions: VG and MP are Chief Investigators and obtained funding for the study. VG and MP led on protocol development with contribution from all named authors in the following ways: PA advised on study design and conduct; MM and PP contributed to MH assessments and analysis; SF lead on physical activity and sleep data collection and analysis; HA lead on economic evaluation; AS advised on the study design and statistical analysis plans; BM developed the statistical analysis plan; KJ advised on school recruitment and data collection procedures and MW and GE lead on PPI. GW, VG and MP drafted the manuscripts and all authors reviewed and approved the final draft.

Funding statement: This project is funded by the NIHR Public Health Research programme (NIHR131396). The views expressed are those of the author(s) and not necessarily those of the NIHR or the Department of Health and Social Care.

Competing interests statement: The authors declare that they have no competing interests.

Word Count: 3903

Acknowledgement and disclaimer: This project is funded by the NIHR Public Health Research programme (NIHR131396). This paper presents independent research supported by the NIHR Birmingham Biomedical Research Centre at the University Hospitals Birmingham NHS Foundation Trust and the University of Birmingham. The views expressed are those of the author(s) and not necessarily those of the NIHR, NHS or the Department of Health and Social Care.

Other acknowledgements: We would like to thank Gareth Evans who is a secondary school teacher and member of the investigator team for advice on study design and conduct. We would also like to thank members of the SMART Schools SSC and DMEC and members of our PPI groups for their advice on the study protocol and conduct.

Patient consent for publication: Not required.

For peer review only - http://bmjopen.bmj.com/site/about/guidelines.xhtml

Provenance and peer review: The SMART Schools Study protocol has received external peer review and been approved through the National Institute for Health Research Public Health Research Programme.

Trial and registration: ISRCTN77948572 and CRN 52232

For peer review only

For peer review only - http://bmjopen.bmj.com/site/about/guidelines.xhtml

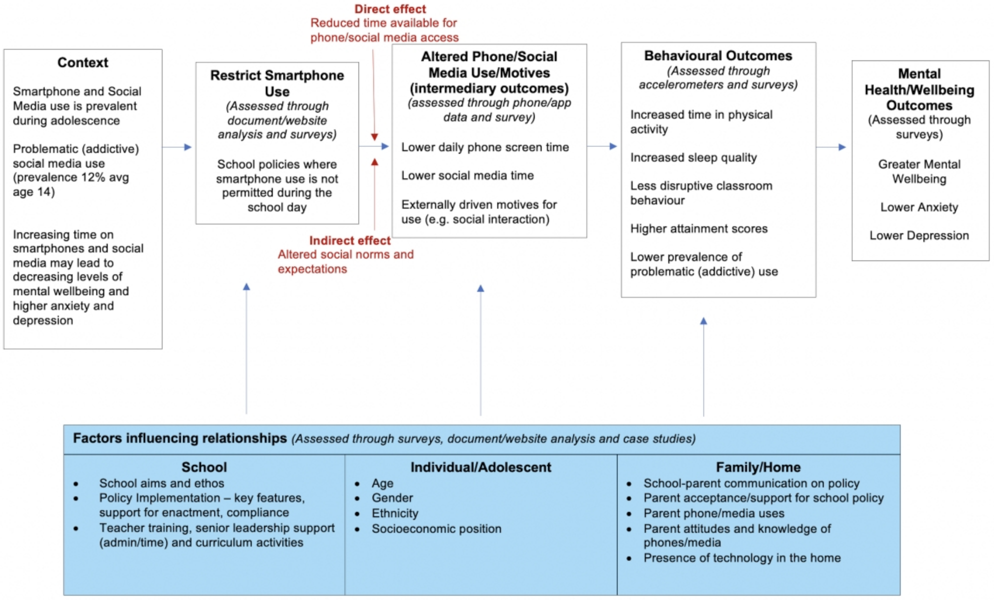

Figure 1. Logic model and theory of change for the influence of school policies that restrict daytime use of smartphones on mental wellbeing and other associated behavioural and health outcomes in adolescents.

411x248mm (300 x 300 DPI)

TIDieR
Template for Intervention Description and Replication

The TIDieR (Template for Intervention Description and Replication) Checklist*:

Information to include when describing an intervention and the location of the information

Item number	Item	Where located **	
		Primary paper (page or appendix number)	Other † (details)
	**BRIEF NAME**		
**1.**	*Name:* SMART Schools Study Smartphones, social Media and Adolescent mental wellbeing: the impact of school policies Restricting dayTime use<br>*Description:* The impact of school daytime restrictions of smartphone and social media use on adolescent mental wellbeing (primary outcome), anxiety, depression, physical activity, sleep, classroom behaviour, attainment, and addictive social media use.	P1, P6	P1 (NHS Health Research Authority (2023) SMART Schools Protocol V6 Available from: https://www.funding[illegible]wards.nihr.ac.uk/aw[illegible]d/NIHR131396)
	**WHY**		
**2.**	*Rationale:* Globally, mental disorders (e.g. anxiety and depression) are the leading cause of disability in adolescents (age range 10-19). At the same time, smartphone and social media use is prevalent during adolescence, and accounts for the majority of their overall screen time. At higher levels of use, time spent on smartphones and social media are associated with decreasing levels of mental wellbeing and higher levels of anxiety and depression.<br><br>There is considerable evidence that school-based interventions can have beneficial effects on adolescent mental wellbeing and associated behavioural outcomes (e.g., sleep, physical activity,	P4-9; Figure 1 on P7, Table 2 on P9	P1-5 (NHS Health Research Authority (2023) SMART Schools Protocol V6. Available from: https://www.fundingawards.nihr.ac.uk/award/NIHR131396)

For peer review only - http://bmjopen.bmj.com/site/about/guidelines.xhtml

classroom behaviour and attainment). School phone policies that restrict daytime phone/media use are an example of a current whole-school environment intervention.

Goal: We suggest that these policies have the potential to lower the overall time adolescents spend on the smartphones/social media, which may improve mental wellbeing and associated behavioural outcomes. There are variations in school-based policies, and these are classified as:

Table 2. Classifications of Variations in School Smartphone Policies as Restrictive (Intervention) and Permissive (Comparator) (P9).

Permissive School Phone Policies (Control)	Restrictive School Phone Policies (Intervention)
Allow pupils to carry phones on them and use them at any time point during the day	Allow phones onto school premises but insist these are not to be used during the school day and are turned off and out of site
Allow pupils to carry phones on them and use them at specific time points during the day (e.g. breaks and lunch)	Allow phones onto school premises, but only allow use of the phones if sanctioned by teaching staff for educational activities (e.g. use of calculator)
Allow pupils to carry phones on them and use them for personal use with consent from school staff	Allow phones onto premises but insist that phones are left in a particular place during the school day e.g. school reception
Allow pupils to carry phones on them and use within designated areas or zones	Pupils are not allowed to carry their phones on school premises all together

Theory: The intervention and data capture are directed by our logic model (Figure 1, P which integrates multiple theories and evidence. First, we adhere to displacement theories to propose that reducing the time adolescents spend on phones/media (i.e. restricting school time use) is optimal for mental wellbeing. Overuse can displace other mental wellbeing promoting activities (e.g. sleep and physical activity) and very low use can deprive adolescents of interactions that support mental wellbeing (e.g., affect and relationships). Second, psychological motives drive phone/media use; motives related to

For peer review only - http://bmjopen.bmj.com/site/about/guidelines.xhtml

	enhancement and social interactions promote mental wellbeing, and motives related to coping and conformity (e.g. Fear of Missing Out) are associated with problematic use (addiction) and poor mental wellbeing. Hence the school policy and ethos have the potential to influence adolescents' motives for using phones/media, which may impact on mental wellbeing. Third, the ecological model of social influence proposes three agents that shape wellbeing and technology use, including the school environment, home/family and individual factors. Finally, policy enactment and implementation process models identify that school policy implementation effects will be shaped by social processes (e.g., training, leadership, compliance, administration, family-school interactions).		
	**WHAT**		
**3.**	*Materials:* Materials for the intervention are context dependent and will vary across schools implementing a restrictive phone policy. Based on our PPI activities, materials may include policy documents; policy communication with parents and adolescents e.g. through school information packs, assemblies, letters and/or the school website.	P7, Table 1 on P8	P7-8, Table 1 on P9 (NHS Health Research Authority (2023) SMART Schools Protocol V6. Available from: https://www.fundingawards.nihr.ac.uk/award/NIHR131396)
**4.**	*Procedures:* Adolescents are not permitted to use their smartphones during lessons or recreational time in the school day, and their smartphones must not be seen in school during these times (except with permission from a teacher)	Table 2 on P9	Table 2 on P10 (NHS Health Research Authority (2023) SMART Schools Protocol V6. Available from: https://www.fundingawards.nihr.ac.uk/award/NIHR131396)

For peer review only - http://bmjopen.bmj.com/site/about/guidelines.xhtml

	**WHO PROVIDED**		
**5.**	Schools develop their own policies (or these are designed by the school's Multi-Academy Trust), and this policy is communicated to all school staff and pupils. Secondary Schools within the sample (see point 7) vary in regions of high and low levels of deprivation and in areas that have high and low proportions of Black, Asian and Minority Ethnic (BAME) groups.	Types of policies presented in Table 2 on P9	Table 2 on P10 (NHS Health Research Authority (2023) SMART Schools Protocol V6. Available from: https://www.fundingawards.nihr.ac.uk/award/NIHR131396)
	**HOW**		
**6.**	*Mode of delivery:* The restrictive school policy is implemented in school contexts, and thus, a face-to-face format. School staff enact the school policy and, in some schools, are required to administer behavioural consequences for adolescents who use their phone during the day, such as: phone confiscation, detention, parent-school meeting. In the intervention schools the restrictive school policy is applied to pupils in year groups 7-11 (age 11-16 years).	Table 2 on P9, Table 3 on P12	Table 1 on P9, Table 2 on P10 (NHS Health Research Authority (2023) SMART Schools Protocol V6. Available from: https://www.fundingawards.nihr.ac.uk/award/NIHR131396)
	**WHERE**		
**7.**	*Where the intervention occurred:* Secondary schools (ages 11-19) within England and located within a 100-mile radius of the recruiting centre in the West Midlands. 64 local authorities are included in the sampling frame from the West Midlands, East Midlands, East, South East, South West and North West. Secondary schools in the sampling frame (including community schools, foundation and voluntary schools, academies, free schools and colleges), had Senior Leadership Team members, school staff and parents/carers capable of giving informed consent, and school pupils aged 12-13 or 14-15 capable of providing assent were included.	P9-11	P12 (NHS Health Research Authority (2023) SMART Schools Protocol V6. Available from: https://www.fundingawards.nihr.ac.uk/award/NIHR131396)

For peer review only - http://bmjopen.bmj.com/site/about/guidelines.xhtml

	Schools other than state-funded mainstream schools (special schools, pupil referral units and independent schools) were excluded because it was expected that there would be additional influences on mental wellbeing. Schools that did not have an accessible smartphone policy and/or had different smartphone policies for the year groups 8 and 10, and schools with missing data that was required for the propensity score estimation were also excluded.		
**8.**	**WHEN and HOW MUCH**<br>Intervention schools in the sample will already have a restrictive policy implemented, hence this study is a natural experiment and observational study. Intervention schools vary in the level of restrictions for phone/media use, with some restrictive school policies permitting pupil phones to be placed in their bags, whereas others do not permit pupils to bring their phones onto the school premises. Intervention schools vary in the duration that the school phone/media policy has been implemented (e.g. <1, 1, 2, or 3> years).	P6-9, Table 2 on P9	P10 (NHS Health Research Authority (2023) SMART Schools Protocol V6. Available from: https://www.fundingawards.nihr.ac.uk/award/NIHR131396)
	**TAILORING**		
**9.**	Schools have developed policies that are tailored to their specific school contexts (or Multi-Academy Trusts). Policies usually apply to the whole school, although in some schools, 6th form pupils (age 16+) may be permitted to use their smartphones during the school day (this age group will not be investigated in this study).	Table 1 on P8,<br>Table 2 on P9	Table 2 on P10 (NHS Health Research Authority (2023) SMART Schools Protocol V6. Available from: https://www.fundingawards.nihr.ac.uk/award/NIHR131396)
**10.**	**MODIFICATIONS**	N/A	

For peer review only

	N/A		
**11.**	**HOW WELL**<br>Adherence: Adherence of the intervention will be captured by the degree to which pupils, school staff and parents adhere to the school policy, and how parents/carers support of the policy varies across schools.	Table 1 on P8, Table 3 on P12	P11 (NHS Health Research Authority (2023) SMART Schools Protocol V6. Available from: https://www.fundingawards.nihr.ac.uk/award/NIHR131396)
**12.**	Actual: N/A	N/A	N/A

** **Authors** - use N/A if an item is not applicable for the intervention being described. **Reviewers** – use '?' if information about the element is not reported/not sufficiently reported.

† If the information is not provided in the primary paper, give details of where this information is available. This may include locations such as a published protocol or other published papers (provide citation details) or a website (provide the URL).

‡ If completing the TIDieR checklist for a protocol, these items are not relevant to the protocol and cannot be described until the study is complete.

* We strongly recommend using this checklist in conjunction with the TIDieR guide (see *BMJ* 2014;348:g1687) which contains an explanation and elaboration for each item.

* The focus of TIDieR is on reporting details of the intervention elements (and where relevant, comparison elements) of a study. Other elements and methodological features of studies are covered by other reporting statements and checklists and have not been duplicated as part of the TIDieR checklist. When a **randomised trial** is being reported, the TIDieR checklist should be used in conjunction with the CONSORT statement (see www.consort-statement.org) as an extension of **Item 5 of the CONSORT 2010 Statement.** When a **clinical trial protocol** is being reported, the TIDieR checklist should be used in conjunction with the SPIRIT statement as an extension of **Item 11 of the SPIRIT 2013 Statement** (see www.spirit-statement.org). For alternate study designs, TIDieR can be used in conjunction with the appropriate checklist for that study design (see www.equator-network.org).

For peer review only - http://bmjopen.bmj.com/site/about/guidelines.xhtml

Smartphones, social Media and Adolescent mental wellbeing: the impact of school policies Restricting dayTime use—protocol for a natural experimental observational study using mixed methods at secondary schools in England (SMART Schools Study)

Journal:	*BMJ Open*
Manuscript ID	bmjopen-2023-075832.R1
Article Type:	Protocol
Date Submitted by the Author:	25-May-2023

Complete List of Authors:	Wood, Grace; University of Birmingham, School of Sport, Exercise and Rehabilitation Sciences; University of Birmingham Institute for Mental Health<br>Goodyear, Victoria; University of Birmingham School of Sport and Exercise Sciences; University of Birmingham Institute for Mental Health<br>Adab, Peymane; University of Birmingham Institute of Applied Health Research, Public Health<br>Al-Janabi, Hareth; University of Birmingham Institute for Mental Health; University of Birmingham Institute of Applied Health Research<br>Fenton, Sally; University of Birmingham School of Sport and Exercise Sciences, School of Sport, Exercise and Rehabilitation Sciences<br>Jones, Kirsty; Services for Education<br>Michail, Maria; University of Birmingham Institute for Mental Health; University of Birmingham School of Psychology<br>Morrison, Breanna; University of Birmingham Institute of Applied Health Research<br>Patterson, Paul; University of Birmingham Institute for Mental Health; Birmingham Women's and Children's NHS Foundation Trust<br>Sitch, Alice; University of Birmingham Institute of Applied Health Research; NIHR Birmingham Biomedical Research Centre, Institute of Translational Medicine<br>Wade, Matthew; ukactive Research Institute; Sheffield Hallam University College of Health Wellbeing and Life Sciences, Advanced Wellbeing Research Centre<br>Pallan, Miranda; University of Birmingham Institute of Applied Health Research

**Primary Subject Heading**:	Mental health
Secondary Subject Heading:	Public health, Epidemiology, Health economics
Keywords:	Adolescent, MENTAL HEALTH, Schools

For peer review only - http://bmjopen.bmj.com/site/about/guidelines.xhtml

Smartphones, social Media and Adolescent mental wellbeing: the impact of school policies Restricting dayTime use—protocol for a natural experimental observational study using mixed methods at secondary schools in England (SMART Schools Study)

Authors: Grace Wood[1,2], Victoria A. Goodyear[1,2*], Peymane Adab[3], Hareth Al-Janabi[2,3], Sally Fenton[1], Kirsty Jones[4], Maria Michail[2,5], Breanna Morrison[3], Paul Patterson[2,6], Alice Sitch[3,7], Matthew Wade[8,9], Miranda Pallan[3].

Affiliations:
[1]School of Sport, Exercise and Rehabilitation Sciences, University of Birmingham, Birmingham, UK
[2]Institute for Mental Health, University of Birmingham, Birmingham, UK,
[3]Institute of Applied Health Research, University of Birmingham, Birmingham, UK
[4]Services for Education, Birmingham, UK
[5]School of Psychology, University of Birmingham, Birmingham, UK
[6]Birmingham Women's and Children's NHS Foundation Trust, Birmingham, UK
[7]NIHR Birmingham Biomedical Research Centre, University Hospitals Birmingham NHS Foundation Trust and University of Birmingham, Institute of Translational Medicine, Birmingham, UK
[8]ukactive, ukactive Research Institute, London, UK
[9]Advanced Wellbeing Research Centre, College of Health, Wellbeing and Life Sciences, Sheffield Hallam University, Sheffield, UK

***Correspondence to:**
Dr Victoria Goodyear
v.a.goodyear@bham.ac.uk

For peer review only

ABSTRACT

Introduction: Smartphone and social media use is prevalent during adolescence, with high levels of use associated with lower levels of mental wellbeing. Secondary schools in the UK have introduced policies that restrict daytime use of smartphones and social media, but there is no evaluation on the impact of these policies on adolescent mental wellbeing. The SMART Schools Study aims to determine the impact of daytime restrictions of smartphone and social media use on indicators of adolescent mental wellbeing, anxiety, depression, physical activity, sleep, classroom behaviour, attainment and addictive social media use.

Methods and analysis: This is a natural experimental, cross-sectional observational study using mixed methods. Secondary schools within a 100-mile radius of the recruiting centre in the West Midlands (UK) have been categorised into two groups: Schools that restrict (intervention) and permit (comparator) daytime use of smartphones. We aim to recruit 30 schools (20 restrictive, 10 permissive) and 1170 pupils aged 12-13 and 14-15 years. We will collect data on mental wellbeing, anxiety and depressive symptoms, phone and social media use, sleep and physical activity from pupil surveys, and accelerometers. Policy implementation measures and data on individual pupil factors will be collected through school staff surveys, and website/policy analysis. Six case study schools will explore individual, school and family/home factors that influence relationships between school smartphone policies, smartphone/social media use, and mental wellbeing. Economic evaluation will be completed through a cost-consequence analysis from an education sector perspective.

Ethics and dissemination: Ethical approval was obtained from the University of Birmingham's Research Ethics Committee (ERN_22-0723). Parents/carers of pupil participants can complete a form to opt their child out of the study. Pupil, school staff and parent/carer participants are asked to complete online/written consent (or assent). Findings will be disseminated through policy briefings, resources for schools, social media, reports, and open access publications.

Study registration: ISRCTN77948572.

Strengths and limitations of this study

- This is a natural experimental observational study exploring the impact of school phone policies that restrict daytime use of smartphones and social media on adolescent mental wellbeing and associated behaviours, and the cost implications of these policies from the education sector perspective.
- Pupils will self-report data on time spent on their smartphones and social media by using data from their iOS or Android apps.
- Pupil outcomes will be collected from; an online survey that includes validated measures for mental wellbeing, anxiety and depressive symptoms, addictive social media use, motives for social media use, health-related quality of life and

For peer review only

demographic data; and from accelerometers measuring physical activity and sleep.

- Qualitative research will explore how individual, school and family/home factors influence relationships between school phone policies, phone/media use and mental wellbeing.
- This is a cross-sectional study and is limited to data collected in England.

KEYWORDS

Smartphones, Social Media, Adolescent Health, Mental Health, Physical Activity, Secondary Schools, School Policy

For peer review only

For peer review only - http://bmjopen.bmj.com/site/about/guidelines.xhtml

INTRODUCTION

Globally, mental disorders (e.g. anxiety and depression) are the leading cause of disability in adolescents (age range 10-19) [1, 2]. In the UK, one fifth of adolescents (20%) are reported to have a mental health disorder [3], mostly anxiety and depression [2]. Half of all mental health disorders start before the age of 14 [4, 5], and if left untreated, mental health problems are highly likely to persist well into adulthood [2, 6]. Poor mental wellbeing also negatively affects other aspects of adolescents' lives, including cognitive, social and physical behaviours [6, 7]. For example, poor mental wellbeing is associated with higher rates of disruptive behaviour, school absence and lower educational attainment [8, 9]. Sleep problems are also common among adolescents diagnosed with anxiety and depression, and evidence suggests a bidirectional relationship between sleep disturbances and mental health problems [10]. Levels of physical activity also decline significantly during adolescence, and this coincides with increased onset of mental health problems [4, 11]. Hence, adolescence is a crucial period for mental health intervention [7], and there is a pressing need to improve and develop approaches to mental health prevention and intervention [6].

Smartphone and social media use is prevalent during adolescence, and accounts for the majority of their overall screen time [12, 13]. In the UK, most adolescents own a smartphone (98%), and are reported to be active users of social media (93%) [14], with comparable trends reported in other Western populations [15]. Samples in the US and the UK (2021-2022) estimate that the time adolescents spend on smartphones and social media ranges between one and a half hours and eight and a half hours per day [12-14, 16, 17], with most adolescents spending between one and three hours per day [14, 16, 17]. Problematic social media use is also prevalent, with 12% of adolescents in England reported to exhibit addictive use behaviours [18].

In moderation, smartphone and social media use (e.g., <2 hours per day) can be advantageous for mental wellbeing and mental health [19-24], as well as other associated health and behavioural outcomes (e.g. sleep, physical activity, classroom behaviour and attainment). However, at higher levels of use, the reverse effect tends to be seen, with increasing time spent on smartphones and social media associated with decreasing levels of mental wellbeing and higher levels of anxiety and depression

For peer review only - http://bmjopen.bmj.com/site/about/guidelines.xhtml
For peer review only

[17, 19-21, 23, 25, 26]. Poor academic performance, disruptive classroom behaviour and less time spent in physical activity and sleep are also more likely in adolescents who spend a greater proportion of time on smartphones and/or social media [19-23, 25, 26]. Reducing the time adolescents spend on smartphones and social media is thus a plausible intervention to improve mental wellbeing, possibly operating through improving the related behavioural outcomes (e.g., physical activity, sleep, academic performance, classroom behaviour). However, uncertainties in the strength of associations between smartphone/social media and mental wellbeing exist, and this is mainly due to reliance on self-reported use [15, 27-29]. Furthermore, individual (e.g. gender, age, socio-economic status) [13, 16, 30-33] and family/home (e.g. parental usage and attitudes toward technology) [34-37] factors are also likely to impact on relationships between smartphone/social media use and mental wellbeing.

There is considerable evidence that school-based interventions can have beneficial effects on adolescent mental wellbeing and associated behavioural outcomes (e.g., sleep, physical activity, classroom behaviour and attainment) [38-41]. Whole-school environment interventions that promote lifestyles conducive to good health are reported to have a more pronounced effect on mental wellbeing than individual approaches targeting knowledge and beliefs [38, 42]. A whole-school approach targets physical and social influences of health, and through the alignment of school policies, values and practices with effective school leadership, the whole school approach seeks to promote a set of values, attitudes and behaviours that encourage the development and maintenance of positive physical, social, cognitive and emotional habits [38, 40]. Evidence suggests that whole school policies related to health and wellbeing can: (i) reduce overall screen time; (ii) positively influence mental wellbeing; and (iii) improve physical activity, sleep, educational attainment and reduce disruptive classroom behaviour [43-45]. Therefore, whole school policies aiming to influence smartphone and social media use have the potential to positively impact on adolescents' mental wellbeing.

School phone policies that restrict daytime phone/media use are an example of a current whole-school environment intervention. In the UK, Australia, Sweden, Czech Republic and elsewhere, many schools have introduced school policies that restrict daytime use of smartphones in order to reduce classroom disruptive behaviour and

For peer review only - http://bmjopen.bmj.com/site/about/guidelines.xhtml

cyberbullying incidents and improve attainment [44, 46, 47]. We suggest that these policies have the potential to lower the overall time adolescents spend on the smartphones/social media, which may improve mental wellbeing and associated behavioural outcomes. However, there is currently no evaluation of the effect of school smartphone policies on mental wellbeing and there is limited evidence on how smartphone policies are implemented in schools [48].

The SMART Schools Study aims to determine the impact of school daytime restrictions of smartphone and social media use on adolescent mental wellbeing (primary outcome), anxiety, depression, physical activity, sleep, classroom behaviour, attainment, and addictive social media use. We will do this by comparing impacts in two different secondary school phone policy contexts: (i) schools that do not permit smartphone use during recreational time in the school day (intervention); and (ii) schools that permit smartphone use during recreational time (breaks/lunchtimes) (comparator). We will also explore how variation in school-based, individual and family/home factors influence the relationship between school phone policies, smartphone and social media use and mental wellbeing. We will conduct an economic evaluation in the form of a cost-consequence analysis from an education sector perspective.

METHODS AND ANALYSIS

This is a natural experimental, cross-sectional observational study using mixed methods, taking place between April 2022 and July 2024. Quantitative and qualitative data will be collected from all schools in the sample (n=30) to compare outcomes between restrictive (intervention) and permissive (comparator) school policies and to complete an economic evaluation. Qualitative data will be collected from six case study schools to understand the contextual factors that could influence relationships between school policies, smartphone and social media use and mental wellbeing.

Intervention: school smartphone policies that restrict daytime use

The intervention and data capture are directed by our logic model (Figure 1) which integrates multiple theories and evidence. First, we adhere to displacement theories

For peer review only - http://bmjopen.bmj.com/site/about/guidelines.xhtml

[19] to propose that reducing the time adolescents spend on phones/media (i.e. restricting school time use) is optimal for mental wellbeing. Overuse can displace other mental wellbeing promoting activities (e.g. sleep and physical activity) and very low use can deprive adolescents of interactions that support mental wellbeing (e.g., affect and relationships) [19, 21, 23]. Second, psychological motives drive phone/media use; motives related to enhancement and social interactions promote mental wellbeing, and motives related to coping and conformity (e.g. Fear of Missing Out) are associated with problematic use (addiction) and poor mental wellbeing [49, 50]. Hence the school policy and ethos have the potential to influence adolescents' motives for using phones/media, which may impact on mental wellbeing. Third, the ecological model of social influence proposes three agents that shape wellbeing and technology use, including the school environment, home/family and individual factors [51]. Finally, policy enactment and implementation process models [39, 52, 53] identify that school policy implementation effects will be shaped by social processes (e.g., training, leadership, compliance, administration, family-school interactions).

Informed by our Patient and Public Involvement (PPI) activities and school smartphone policy analysis, the components of the intervention (i.e., restrictive school smartphone policies) are presented in Table 1. In Table 2 we have outlined variations in school smartphone policies and our classification of these variations into two school policy groups: restrictive (intervention) and permissive (comparator).

Table 1. Components of the intervention, guided by the template for intervention description and replication (TIDieR) checklist [54]

TiDieR components	SMART Schools intervention
Description	School policy prohibiting the use of smartphones during the school day
Materials	The policy may be communicated to parents/carers and adolescents in a variety of ways, such as through school information packs, assemblies, letters and/or the school website
Procedures	Adolescents are not permitted to use their smartphones during lessons or recreational time in the school day, and their smartphones must not be seen in school during these times
Provider	Schools (or Multi-Academy Trusts*) develop their own policies,

For peer review only - http://bmjopen.bmj.com/site/about/guidelines.xhtml

	often in consultation with school staff, parents and/or school governors, and in relation to the school ethos
Mode of Delivery	The Senior Leadership team (SLT) and school staff enact the school policy and are required to administer behavioral consequences for adolescents who use their smartphone during the day, such as smartphone confiscation, detention and/or parent-school meeting
Time Period	Schools vary in terms of how long their school smartphone has been implemented
Tailoring	Schools have developed policies according to their specific school contexts (or Multi-Academy Trusts*). Policies usually apply to the whole school, although in some schools 6th form pupils (age 16+) may be permitted to use their smartphones during the school day (this age group will not be investigated in this study)
Adherence and Fidelity	The degree to which pupils and teachers adhere to the school policy, and parents/carers are in support of the policy varies across schools

* Multi-Academy Trusts (MAT) –non-profit companies that manage more than one academy [55].

Table 2. Classifications of variations in school smartphone policies as restrictive (intervention) and permissive (comparator)

Restrictive school smartphone policies (intervention)	Permissive school smartphone policies (comparator)
Allow smartphones onto school premises but insist these are not to be used during the school day and are turned off and out of sight	Allow pupils to carry smartphones and use them at any time point during the day
Allow smartphones onto school premises, but only allow use if sanctioned by teaching staff for educational activities (e.g., use of calculator)	Allow pupils to carry smartphones and use them at specific time points during the day (e.g., breaks and lunch)
Allow smartphones onto premises but insist they are left in a specified place	Allow pupils to carry smartphones and use them for personal use with consent from school staff

For peer review only - http://bmjopen.bmj.com/site/about/guidelines.xhtml

during the school day e.g., school reception or lockers	
Pupils are not allowed to carry their smartphones onto school premises at any time	Allow pupils to carry smartphones and use within designated areas or zones

Study setting

The sampling frame comprises UK secondary schools (ages 11-19) located within a 100-mile radius of the recruiting centre in the West Midlands. 64 local authorities are included in the sampling frame from the West Midlands, East Midlands, East, South East, South West and North West. The schools are situated in regions of high and low levels of deprivation and in areas that have high [56] and low proportions of Black, Asian and Minority Ethnic (BAME) groups [57, 58]. Schools other than state-funded mainstream schools (special schools, pupil referral units and independent schools) were excluded because it was expected that there would be additional influences on mental wellbeing. Schools that did not have an accessible smartphone policy and/or had different smartphone policies for the year groups 8 and 10, and schools with missing data that was required for the propensity score estimation (see sampling and participants) were also excluded (n=10,810). A total of 1345 secondary schools are included in our sampling frame; 1220 schools with policies classified as restrictive (intervention) and 125 as permissive (comparator).

Sampling and participants

To improve the comparability of the two school groups, stratified sampling based on propensity scores was employed [59]. We obtained routine data from the Department for Education on the following school characteristics: region; school type; urban or rural; total pupil roll size; Income Deprivation Affecting Children Index (IDACI) [60]; inclusion of a sixth form; selective or non-selective admissions policy; religious affiliation; and the proportion of pupils with the following characteristics: male; from BAME groups; English as an Additional Language; eligible for free school meals; and Special Education Needs. Propensity scores were calculated using restrictive or permissive school smartphone policies as the outcome and school characteristics as

For peer review only - http://bmjopen.bmj.com/site/about/guidelines.xhtml

explanatory variables. Propensity score terciles were then used to create three groups with subsequent division by restrictive or permissive policy type, resulting in 6 distinct sampling groups. Schools in each group have been randomly ordered and are being invited sequentially to participate, aiming to recruit six to seven schools from each restrictive tercile and three to four from each permissive tercile to achieve a sample size of 20 schools with restrictive and 10 schools with permissive policies.

In participating schools, two classes of mixed ability year 8 (age 12-13) and year 10 (age 14-15) pupils are recruited. We are focusing on adolescents in this age range because of the age of onset and prevalence of mental illness [5], and the prevalence of smartphone and social media use by adolescents aged 12-15 [7, 14, 61]. Within this age range we may also be able to observe potential differences in smartphone/ social media use relationships with wellbeing [33]. For example, year 8 pupils are likely to be newer users of phones/media and physical activity levels begin to decline at this age, particularly among girls [7, 13]. In year 10, pupils are more likely to be established phone/media users, mental wellbeing tends to be lower, and this age group are approaching the peak onset of mental health conditions [4]. In addition to adolescent participants, the form teacher for each class recruited (or an equivalent teacher responsible for the class) and a member of SLT who is responsible for the school smartphone policy are recruited from each school.

Sample size calculation

To account for the imbalance of schools in our sample that have permissive (n=125) and restrictive policies (n=1220), we are recruiting schools using a 2:1 (restrictive: permissive) ratio. The primary outcome of mental wellbeing will be measured using the Warwick-Edinburgh Mental Well-Being Scale (WEMWBS; score range=14-70) [62]. To detect a mean difference in score of three points (considered the minimum clinically important difference [62] between the 2 school groups), assuming a SD of 6.8 [63] and an ICC of 0.1 (a conservative estimate [64]), with 90% power and 5% significance, we require 20 schools in the restrictive and 10 schools in the permissive smartphone policy groups, with an average cluster size of 39 (1170 pupil participants in total; 780 in the restrictive, and 390 in the permissive policy groups).

For peer review only - http://bmjopen.bmj.com/site/about/guidelines.xhtml

In each participating class, we aim to recruit a minimum of 19-20 pupils (67% if estimated class size n=30). In studies with multiple layers of clustering (here classes within schools), it is conservative to treat clusters within clusters as one larger cluster, which is the approach used here [65].

Recruitment

School recruitment commenced in September 2022 and will continue until December 2023. A study advertisement has been emailed to all schools in the sampling frame. Following our propensity sampling approach, schools are then invited by post and email with a telephone follow-up. In participating schools, a School Liaison Member of staff (SLM) is identified and a school-university contract outlining expected commitments signed. Subsequently, a member of SLT responsible for the smartphone policy, and pupils and teachers from the year 8 and year 10 classes are recruited. £600 compensation is allocated to each school and a £5 voucher per pupil participant. Parents/carers are provided with written detailed information about the study, what their child's participation will involve and how their child's data will be processed. Schools are asked to assist in the distribution of this information to parents/carers in different formats (e.g., email, post, text messages, website etc.).

Data collection

Data collection methods include self-administered surveys for pupils, teachers and a member of the SLT; accelerometer measured physical activity and sleep for pupils; and document analysis. All online surveys are completed using university-approved online survey software (REDCap). Table 3 provides an overview of outcomes, measures and timepoints of evaluation.

Table 3. Overview of outcomes, measures and timepoints of evaluation

Outcome	Outcome measures	Timepoint(s) of evaluation
**Primary outcome**		
Mental wellbeing	Warwick-Edinburgh Mental Well-Being Scale (WEMWBS) [62] / pupil survey	Measured over the past 2 weeks

For peer review only

		at 2 time points, 4-8 weeks apart
**Secondary outcomes**		
Anxiety symptoms	Generalized Anxiety Disorder Assessment (GAD-7) [66] / pupil survey	1 time point measured over the past 2 weeks
Depressive symptoms	Patient Health Questionnaire (PHQ-9) [67] / pupil survey	1 time point measured over the past 2 weeks
Addictive use	Problematic Social Media use Scale [49, 50] / pupil survey	1 time point based on usual use
Sleep	Sleep quality (indicated by sleep duration and sleep efficiency / accelerometers<br><br>Time pupils go to sleep and wake time on a school day and a weekend day / pupil survey	Over 7 days / usual behaviours
Physical activity	Physical activity (total and moderate-to-vigorous intensity (MVPA) / accelerometers<br><br>Active travel and sports/activity club engagement/ pupil survey	Over 7 days / usual behaviours<br><br>Usual behaviours
Attainment	Assessment of whether pupils are below, above or working at their target grade in English and Maths / teacher survey	1 time point – most recent assessment
Disruptive classroom behaviour	Pupil Behaviour Questionnaire [68] / teacher survey	1 time point – current assessment
**Intermediate outcomes**		
Smartphone use duration	3 measures: (i) within school; (ii) over 24-hour period on a school day; (iii) on a weekend day / data captured from iOS and Android smartphones and will be self-reported through the pupil survey	1 time point
Social media use duration	3 measures: (i) within school; (ii) over 24-hour period on a school day; (iii) on a weekend day / data captured from iOS and Android smartphones and will be self-reported through the pupil survey	1 time point
Motives for social media use	Social Media Motives Questionnaire / pupil survey	1 time point measured over past 12 months
**Policy implementation measures**		

For peer review only - http://bmjopen.bmj.com/site/about/guidelines.xhtml

Intervention components (school level data): school timetable; school policies (smartphone, mental health, behaviour, e-safety): school smartphone policy details (e.g., rules/key features; time period of implementation; policy communication and understanding; adherence and fidelity and policy rationale)	School policy documents<br>School handbooks<br>School website content<br>SLT survey<br>Teacher survey<br>Pupil survey	1 time point
Individual factors (pupils): age, gender, ethnicity, socio-economic position, eligibility for Free School Meals, and whether pupils have English as an additional language and/or special educational needs	Pupil survey<br>Teacher survey	1 time point
Contextual factors: school, individual, family/home	Focus groups with school staff, pupils and parents/carers of school pupils	1 time point
**Economic variables**		
Teacher time spent managing policy<br>Indirect time spent managing behavioural problems<br>Other related costs	SLT Survey<br>Teacher survey	1 time point
Health-related quality of life / Quality Adjusted Life Years	The Child Health Utility Instrument (CHU9D) [69] / pupil survey	1 time point / current assessment

Pupil online survey

Pupils complete an online survey at one time point (in school time) that includes validated measures for mental wellbeing, anxiety and depressive symptoms, addictive social media use, motives for social media use, health-related quality of life and demographic variables (e.g., age, gender) (Table 3). Within the survey pupils are asked to self-report data on their physical activity levels, sleep and phone/media use, and by using data from their iOS or Android apps, they additionally self-report data on time spent on their smartphone and social media apps (Table 3). Within the survey pupils are also asked to report on their knowledge and understanding of the school

For peer review only - http://bmjopen.bmj.com/site/about/guidelines.xhtml

smartphone policy and compliance with the school smartphone policy The survey is completed on encrypted tablets, using a portable Wi-Fi hub owned by the research team.

Secondary measure of mental wellbeing

A second online survey (in school time) to measure mental wellbeing (Table 3) is completed four to eight weeks after pupils have completed the initial online survey. The SLM is sent an email link for pupils to complete.

Teacher online survey

Data are collected from the form teacher (or an equivalent teacher responsible for the class) for each participating pupil on: pupil attainment; classroom behavior; and whether pupils are eligible for free school meals, have a special educational need, or have English as an additional language (Table 3). Within the survey teachers are also asked to report on their knowledge and understanding of the school smartphone policy, compliance with the school smartphone policy and to report on the time they spend implementing the school smartphone policy (Table 3). Teachers are sent the online survey by email.

Senior Leadership Team online survey

The SLT member responsible for the school smartphone policy is asked to complete survey questions on: the SLT member's role; the school timetable and school policies; features of the school smartphone policy; perceived time spent by school staff developing and implementing the school smartphone policy; knowledge and understanding of the school smartphone policy; and compliance with the school smartphone policy. The SLT member is sent the online survey by email.

Accelerometers

Pupils are asked to wear a wrist worn GENEActiv accelerometer watch for 24 hours a day for the subsequent seven days after completing the survey [70]. Watches are worn on the non-dominant wrist during all activities, including water-based activities.

For peer review only - http://bmjopen.bmj.com/site/about/guidelines.xhtml

Accelerometers are initialised to collect data in 100Hz. Data will be analysed in R to produce physical activity and sleep outcomes (Table 3).

Document analysis

School policy documents and handbooks related to smartphones, social media, pupil behavior, mental health/wellbeing, e-safety/IT policy and the wider school aims and ethos are collected. Documents related to the school timetable are also collected so that time spent in physical education, and time allocated for breaks and lunch can be quantified for each school.

Case study schools

Six case study schools are being purposively sampled from the 30 participating schools in relation to two school characteristics: (i) smartphone policy type and duration to ensure a balance of restrictive (n=3) vs permissive policies (n=3), and a range of length of policy implementation in the six case studies; and (ii) schools from low, medium and high area of deprivation, measured by the Income Deprivation Affecting Children Index (IDACI). An additional £300 compensation is allocated to case study schools.

Across the case study schools, up to 36 focus groups (FG) will be completed with adolescents (n=12), school staff (n=12) and parents/carers (n=12). Data collection is taking place following the school level data collection (i.e., surveys, accelerometers and document analysis) and following obtaining written consent (or assent). In each case study school four to six FGs are completed: adolescents (n=2), school staff (n=1-2) and parents/carers (n=1-2). Each FG comprises four to six members and aims to balance gender and ethnicity (where possible). For adolescents, one FG is completed with year 8 (age 12-13) and one with year 10 (age 14-15) pupils. School staff FGs include SLT, school governors, teachers and support staff (admin, caretakers, teaching assistants). Parents/carers of pupils from within the school (excluding 6th form, age 16-19) are recruited.

For peer review only - http://bmjopen.bmj.com/site/about/guidelines.xhtml

FGs are led by research staff employing established elicitation and semi-structured interview techniques [71, 72]. Each FG lasts approximately 60 minutes and takes place in school or online. Data are collected from voice transcription.

Data analysis

Analysis of pupil outcomes

The primary analysis will examine the association between pupil mental wellbeing and school policy type (restrictive/permissive). Multilevel linear models will be developed, accounting for repeated measures, clustering of classes and schools and adjusting for the school-level variables included in the propensity score estimations alongside relevant individual-level sociodemographic variables. Secondary analyses will use the same modelling approach investigating differences in the secondary (behavioural and mental health) and intermediary (smartphone and social media use) outcomes between school policy groups. Differential association between school policy and the primary, secondary and intermediary outcomes will also be explored across: (i) socioeconomic position; (ii) gender; and (iii) ethnicity by including relevant interaction terms in the developed models.

Analysis of school policies, documents and websites

School policies, documents (e.g. school handbook) and relevant website content will be analysed using document analysis [73]. This will provide an overview of smartphone policy content, rationale and communication, and how schools support pupils to use their smartphones and social media, as well as their mental wellbeing. We will adopt a comparative approach to compare restrictive and permissive policies.

Economic analysis

In view of the multiple outcomes of interest, complex nature of school budgets and emergent nature of economic evaluation of school-based interventions, a cost-consequence analysis will be conducted through the relevant data collected (Table 3). This approach has been previously employed for school-based interventions [74]. In

For peer review only - http://bmjopen.bmj.com/site/about/guidelines.xhtml

addition to the WEMWBS and CHU-9D outcomes, secondary behavioural and health outcomes outlined in the intervention logic model will be included (Figure 1). An exploratory cost-utility analysis from the payer (school) perspective will be conducted to compare incremental education costs and incremental Quality-Adjusted Life-Years associated with restrictive daytime smartphone use.

Qualitative/case study analysis

Given the purpose of the case study method to understand complexity and situatedness [75], coupled with the overarching aim of this study to compare schools that have restrictive and permissive smartphone policies, it seems appropriate to analyse the case study data taking a dual approach. Thus, each case study will be analysed individually using a thematic analysis [76], and then multiple case analysis will be adopted [77]. Following this approach, the findings will then be reported in detailed 'vertical' case reports on single schools, and in multiple case thematic analysis [77].

Patient and public involvement

During research plan development, a teacher member of the investigator team advised on the study. We also consulted with adolescents and teachers through an online survey (teachers n=40) and five FG consultations (teachers n=11; adolescents n=20). In addition, we undertook analysis of school websites to determine the nature of school smartphone policies. Information gathered through these activities helped to inform and refine the study design, including categorization of school policy types, research questions, data collection methods, primary and secondary outcomes and the logic model (Figure 1).

The approach to PPI during the SMART Schools Study has been constructed based on National Institute for Health Research guidelines [78, 79]. We are engaging with two groups throughout the study: (i) adolescents (from secondary schools); and (ii) adults (from schools/teachers, parents/families, local/national health organizations and policy). These groups are directly impacted by the research and are representative of key stakeholders who would act on the findings. We have one PPI Lead and a PPI Co-applicant, who are leading on PPI activities. Throughout the study

For peer review only - http://bmjopen.bmj.com/site/about/guidelines.xhtml

there will be four online meetings per group which will focus on: (i) designing and managing study procedures; (ii) undertaking the research; and (iii) dissemination. The results and conclusions from each PPI group meeting will be reported using Guidance for Reporting Involvement of Patients and the Public (GRIPP2) checklist [80] for reporting PPI in research.

ETHICS AND DISSEMINATION

Ethical and regulatory considerations

Full ethical approval was obtained from the University of Birmingham's Science Technology, Engineering and Mathematics Research Ethics Committee on 8th July 2022 (ERN_22-0723).

For participation in the study, parents/carers of pupil participants are not asked for active consent but are given the opportunity to complete and return a form to opt their child out of taking part in the study. Pupil, teacher, SLT and parent/carer participants are asked to provide online or written consent (or assent).

We have also developed a safeguarding protocol for conducting research on mental health with adolescents. This safeguarding protocol has been developed in the context of our measure for depression (PHQ-9) that detects whether adolescents in our sample may have had self-harm of suicidal thoughts in the past two weeks. The protocol outlines key procedures during recruitment, data collection and de-briefing periods to safeguard pupils, and include steps related to confidentiality and establishing efficient, secure and effective communication pathways between the research team and school leaders.

Study sponsorship is provided by the University of Birmingham, with provision of research related costs supported by the National Institute for Health Research (NIHR). Data management and storage is compliant with the University of Birmingham's policies and procedures. Participant data from online surveys and other sources will be pseudo-anonymised, stored on a University of Birmingham secure server and

For peer review only - http://bmjopen.bmj.com/site/about/guidelines.xhtml

retained for 10 years through the University's Research Data Archive. Data from this study will be owned by the University of Birmingham.

Study oversight will be guided by an independently chaired Study Steering Committee (SSC) and a Data Management and Ethics Committee (DMEC). A study monitoring plan has been developed and agreed upon by the SSC and DMEC. The current protocol has been reviewed and agreed upon by all members of the SSC and DMEC.

Dissemination

Dissemination activities will be co-produced with our PPI participants. We have planned the following dissemination outputs: Policy briefings and research summaries for agencies on the impact of school smartphone policies on adolescent mental wellbeing; Guidelines and resources for schools on the characteristics of school smartphone and social media policy implementation that positively influence mental wellbeing; Blogs, podcasts, videos and infographics to raise awareness and understanding of relationships between smartphones and social media and mental wellbeing; an NIHR public report that summarises the main project findings; and peer review and open access publications focused on the main study findings.

For peer review only

Contributors: VG and MP are Chief Investigators and obtained funding for the study. VG and MP led on protocol development with contribution from all named authors in the following ways: PA advised on study design and conduct; MM and PP contributed to MH assessments and analysis; SF lead on physical activity and sleep data collection and analysis; HA lead on economic evaluation; AS advised on the study design and statistical analysis plans; BM developed the statistical analysis plan; KJ advised on school recruitment and data collection procedures and MW led on PPI. GW, VG and MP drafted the manuscripts and all authors reviewed and approved the final draft.

Funding: This project is funded by the NIHR Public Health Research programme (NIHR131396). The views expressed are those of the author(s) and not necessarily those of the NIHR or the Department of Health and Social Care.

Competing interests: The authors declare that they have no competing interests.

Word count: 4065

Disclaimer: This project is funded by the NIHR Public Health Research programme (NIHR131396). This paper presents independent research supported by the NIHR Birmingham Biomedical Research Centre at the University Hospitals Birmingham NHS Foundation Trust and the University of Birmingham. The views expressed are those of the author(s) and not necessarily those of the NIHR, NHS or the Department of Health and Social Care.

Acknowledgements: We would like to thank Gareth Evans, who is a secondary school teacher and member of the investigator team, for advice on study design and conduct. We would also like to thank members of the SMART Schools SSC and DMEC and members of our PPI groups for their advice on the study protocol and conduct.

Patient consent for publication: Not required.

Provenance and peer review: The SMART Schools Study protocol has undergone external peer review via the study funder and been approved through the National Institute for Health Research Public Health Research Programme.

For peer review only

REFERENCES

1. World Health Organization. Adolescent Health [online] 2022. https://www.who.int/health-topics/adolescent-health#tab=tab_1 (Accessed 23rd February 2022).
2. World Health Organization. Health for the World's Adolescents - A Second Change in the Second Decade. Geneva, Switzerland; 2014.
3. NHS Digital. Mental Health of Children and Young People in England 2022- wave 3 follow up to the 2017 survey [online] 2022. https://digital.nhs.uk/data-and-information/publications/statistical/mental-health-of-children-and-young-people-in-england/2022-follow-up-to-the-2017-survey# (Accessed 23rd February 2022).
4. Kessler RC, Amminger GP, Aduilar-Gaxiola S, et al. Age of onset of mental disorders: a review of recent literature. Curr Opin Psychiatry 2007; 20(4):359-64.
5. Kessler RC, Berglund P, Demler O, et al. Lifetime prevalence and age-of-onset distributions of DSM-IV disorders in the National Comorbidity Survey Replication. Arch Gen Psychiatry 2005; 62(6):593-602.
6. Colizzi M, Lasalvia A, Ruggeri M. Prevention and early intervention in youth mental health: is it time for a multidisciplinary and trans-diagnostic model for care? IJMHS 2020;14(1):23.
7. Patton GC, Sawyer SM, Santelli JS, et al. Our future: a *Lancet* commission on adolescent health and wellbeing. The Lancet 2016;387(10036):2423-78.
8. Finning K, Ukoumunne OC, Ford T, et al. The association between child and adolescent depression and poor attendance at school: A systematic review and meta-analysis 2019; 245:928-38.
9. Veldman K, Bültmann U, Stewart RE, et al. Mental health problems and educational attainment in adolescence: 9-year follow-up of the TRAILS study. PLoS One 2014;9(7):e101751.
10. Orchard F, Gregory AM, Gradisar M, et al. Self-reported sleep patterns and quality amongst adolescents: cross-sectional and prospective associations with anxiety and depression. J Child Psychol Psychiatry 2020;61(10):1126-37.
11. Zimmermann-Sloutskis D, Wanner M, Zimmermann E, et al. Physical activity levels and determinants of change in young adults: a longitudinal panel study. International Journal of Behavioral Nutrition and Physical Activity 2010;7(1):2.
12. Rideout V, Peebles A, Mann S, et al. Common Sense census: Media use by tweens and teens, 2021. San Francisco, CA; 2022.
13. Ofcom. Children and parents: media use and attitudes report 2022 [online]. 2022. https://www.ofcom.org.uk/__data/assets/pdf_file/0024/234609/childrens-media-use-and-attitudes-report-2022.pdf (Accessed 23rd February 2022).
14. Ofcom. Children and Parents: Media Use and Attitudes 2023 [online] 2023. https://www.ofcom.org.uk/__data/assets/pdf_file/0027/255852/childrens-media-use-and-attitudes-report-2023.pdf (Accessed 31st March 2023).
15. Orben A. Teenagers, screens and social media: a narrative review of reviews and key studies. Social Psychiatry and Psychiatric Epidemiology. 2020;55(4):407-14.
16. Scott H, Biello SM, Woods HC. Social media use and adolescent sleep patterns: cross-sectional findings from the UK millennium cohort study. BMJ Open 2019;9(9):e031161.
17. Plackett R, Sheringham J, Dykxhoorn J. The Longitudinal Impact of Social Media Use on UK Adolescents' Mental Health: Longitudinal Observational Study. J Med Internet Res 2023;25:e43213.

For peer review only - http://bmjopen.bmj.com/site/about/guidelines.xhtml

18. Brooks F KE, Chester K, Magnusson J, et al. Health Behaviour in School-aged Children (HSBC): World Health Organization Collaborative Cross National Study. Findings from the 2018 HSBC study for England. Hatfield, England; 2020.
19. Przybylski AK, Weinstein N. A Large-Scale Test of the Goldilocks Hypothesis. Psychol Sci 2017;28(2):204-15.
20. Przybylski AK, Orben A, Weinstein N. How Much Is Too Much? Examining the Relationship Between Digital Screen Engagement and Psychosocial Functioning in a Confirmatory Cohort Study. J Am Acad Child Adolesc Psychiatry 2020;59(9):1080-8.
21. Viner RM, Gireesh A, Stiglic N, et al. Roles of cyberbullying, sleep, and physical activity in mediating the effects of social media use on mental health and wellbeing among young people in England: a secondary analysis of longitudinal data. Lancet Child Adolesc Health 2019;3(10):685-96.
22. Yan H, Zhang R, Oniffrey TM, et al. Associations among Screen Time and Unhealthy Behaviors, Academic Performance, and Well-Being in Chinese Adolescents. Int J Environ Res Public Health 2017;14(6).
23. Royal College of Psychiatrists. Technology use and the mental health of children and young people [online] 2020. https://www.rcpsych.ac.uk/docs/default-source/improving-care/better-mh-policy/college-reports/college-report-cr225.pdf (Accessed 23rd February 2022).
24. Dienlin T, Johannes N. The impact of digital technology use on adolescent well-being. Dialogues in Clinical Neuroscience 2020;22(2):135-42.
25. Kelly Y ZA, Booker C, Sacker A. Social Media Use and Adolescent Mental Health: Findings From the UK Millennium Cohort Study. eClinicalMedicine 2019;6:59-68.
26. Twenge JM, Martin GN, Campbell WK. Decreases in psychological well-being among American adolescents after 2012 and links to screen time during the rise of smartphone technology. Emotion 2018;18(6):765-80.
27. Orben A, Przybylski AK. Screens, Teens, and Psychological Well-Being: Evidence From Three Time-Use-Diary Studies. Association for Psychological Science 2019;30:682–96.
28. Kandola A, Owen N, Dunstan DW, et al. Prospective relationships of adolescents' screen-based sedentary behaviour with depressive symptoms: the Millennium Cohort Study. Psych Med 2022;52(15):3531-9.
29. Sewall CJR, Bear TM, Merranko J, et al. How psychosocial well-being and usage amount predict inaccuracies in retrospective estimates of digital technology use. Mobile Media & Communication 2020;8(3):379-99.
30. Patalay P, Fitzsimons E. Development and predictors of mental ill-health and wellbeing from childhood to adolescence. Soc Psychiatry Psychiatr Epidemiol 2018;53(12):1311-23.
31. Sport England. Active Lives Children and Young People Survey: Academic Year 2021-22 [online] 2022. https://sportengland-production-files.s3.eu-west-2.amazonaws.com/s3fs-public/2022-12/Active%20Lives%20Children%20and%20Young%20People%20Survey%20Academic%20Year%202021-22%20Report.pdf?VersionId=R5_hmJHw5M4yKFsewm2vGDMRGHWW7q3E (Accessed 23rd February 2022).
32. NHS. Mental Health of Children and Yougn People in England, 2017 [PAS] [online] 2018. https://digital.nhs.uk/data-and-

For peer review only - http://bmjopen.bmj.com/site/about/guidelines.xhtml

information/publications/statistical/mental-health-of-children-and-young-people-in-england/2017/2017 (Accessed 23rd February 2022).
33. Orben A, Przybylski AK, Blakemore S-J, et al. Windows of developmental sensitivity to social media. Nature Communications 2022;13(1):1649.
34. Roffery S. Positive Relationships. London: Springer; 2012.
35. Salway RE, Emm-Collison L, Sebire S, et al. Associations between socioeconomic position and changes in children's screen-viewing between ages 6 and 9: a longitudinal study. BMJ Open 2019;9(12):e027481.
36. Thompson JL, Sebire SJ, Kesten JM, et al. How parents perceive screen viewing in their 5–6 year old child within the context of their own screen viewing time: a mixed-methods study. BMC Public Health 2017;17(1):471.
37. Solomon-Moore E, Matthews J, Reid T, et al. Examining the challenges posed to parents by the contemporary screen environments of children: a qualitative investigation. BMC Pediatrics 2018;18(1):129.
38. Bonell C, Jamal F, Harden A, et al. Public Health Research. Systematic review of the effects of schools and school environment interventions on health: evidence mapping and synthesis. Southamptom: NIHR Journals Library; 2013.
39. Paulus FW, Ohmann S, Popow C. Practitioner Review: School-based interventions in child mental health. JCPP 2016;57(12):1337-59.
40. Glazzard J. A whole-school approach to supporting children and young people's mental health. JPMH 2019;18(4):256-65.
41. Barker R, Hartwell G, Egan M, et al. The importance of school culture in supporting student mental health in secondary schools. Insights from a qualitative study. British Educational Research Journa 2023;00:1-23.
42. Leatherdale ST, Faulkner G, Arbour-Nicitopoulos K. School and student characteristics associated with screen-time sedentary behavior among students in grades 5-8, Ontario, Canada, 2007-2008. Prev Chronic Dis 2010;7(6):A128.
43. Katapally TR, Laxer RE, Qian W, et al. Do school physical activity policies and programs have a role in decreasing multiple screen time behaviours among youth? Prev Med 2018;110:106-13.
44. Kvardova N VN. Do mobile phone bans work? Rules and the use of technology in school in relation to adolescents' behaviour. Research Report from primary and secondary schools. Brno: Masaruk University; 2019.
45. Dix KL, Slee PT, Lawson MJ, et al. Implementation quality of whole-school mental health promotion and students' academic performance. Child Adolesc Ment Health 2012;17(1):45-51.
46. Selwyn N, Aagaard J. Banning mobile phones from classrooms—An opportunity to advance understandings of technology addiction, distraction and cyberbullying. British Journal of Educational Technology 2021;52(1):8-19.
47. Beland L-P, Murphy R. Ill Communication: Technology, distraction & student performance. Labour Economics 2016;41:61-76.
48. House of Commons. Impact of social media and screen-use on young people's health. London: House of Commons; 2019.
49. Marino C, Vieno A, Altoè G, et al. Factorial validity of the Problematic Facebook Use Scale for adolescents and young adults. J Behav Addict. 2017;6(1):5-10.
50. Marino C, Mazzieri E, Caselli G, et al. Motives to use Facebook and problematic Facebook use in adolescents. J Behav Addict 2018;7(2):276-83.
51. Livingstone S, Sefton-Green J. The Class: Living and Learning in the Digital Age. New York: New York University Press; 2016.
52. Ball S. Education Debate. London: Policy Press; 2017.

For peer review only - http://bmjopen.bmj.com/site/about/guidelines.xhtml

53. Ball SJ, Maguire M, Braun A. How schools do policy. London: Routledge; 2012.
54. Hoffmann TC, Glasziou PP, Boutron I, et al. Better reporting of interventions: template for intervention description and replication (TIDieR) checklist and guide. BMJ 2014;348:g1687.
55. Haves E. Education: multi academy trusts [online] 2022. https://lordslibrary.parliament.uk/education-multi-academy-trusts/ (accessed 31st March 2022).
56. Office for National Statistics. Mapping income deprivation at a local authority level [online] 2021. https://www.ons.gov.uk/peoplepopulationandcommunity/personalandhouseholdfinances/incomeandwealth/datasets/mappingincomedeprivationatalocalauthoritylevel (Accessed 23rd February 2022).
57. Office for National Statistics. Ethnicity and National Identity 2012 [online] 2012. https://www.ons.gov.uk/peoplepopulationandcommunity/culturalidentity/ethnicity/articles/ethnicityandnationalidentityinenglandandwales/2012-12-11#:~:text=The%20West%20Midlands%20had%20a,British%20at%2079.2%20per%20cent (Accessed 23rd February 2022).
58. Office for National Statistics. Population Characteristics Research Tables 2019 [online] 2019. https://www.ons.gov.uk/peoplepopulationandcommunity/populationandmigration/populationestimates/datasets/populationcharacteristicsresearchtables (Accessed 23rd February 2022).
59. Rubin DB. Estimating causal effects from large data sets using propensity scores. Ann Intern Med 1997;127(8 Pt 2):757-63.
60. Ministry of Housing, Communities & Local Government. The English Indices of deprivation 2019 (IoD2019). London: Ministry of Housing, Communities & Local Government; 2019.
61. Ofcom. Children and parents: Media use and attitudes report [online] 2019. https://www.ofcom.org.uk/__data/assets/pdf_file/0023/190616/children-media-use-attitudes-2019-report.pdf (Accessed 23rd February 2022).
62. Warwick Medical School. Collect, score, analuse and interpret WEMWBS [online] 2020. https://warwick.ac.uk/fac/sci/med/research/platform/wemwbs/using/howto/ (Accessed 23rd February 2022).
63. Clarke A, Friede T, Putz R, et al. Warwick-Edinburgh Mental Well-being Scale (WEMWBS): Validated for teenage school students in England and Scotland. A mixed methods assessment. BMC Public Health 2011;11(1):487.
64. Hale DR, Patalay P, Fitzgerald-Yau N, et al. School-level variation in health outcomes in adolescence: analysis of three longitudinal studies in England. Prev Sci 2014;15(4):600-10.
65. Hemming K, Lilford R, Girling AJ. Stepped-wedge cluster randomised controlled trials: a generic framework including parallel and multiple-level designs. Stat Med 2015;34(2):181-96.
66. Löwe B, Decker O, Müller S, et al. Validation and standardization of the Generalized Anxiety Disorder Screener (GAD-7) in the general population. Med Care 2008;46(3):266-74.
67. Kroenke K, Spitzer RL, Williams JB. The PHQ-9: validity of a brief depression severity measure. J Gen Intern Med 2001;16(9):606-13.
68. Allwood M, Allen K, Price A, et al. The reliability and validity of the pupil behaviour questionnaire: a child classroom behaviour assessment tool. Emotional and Behavioural Difficulties 2018;23(4):361-71.

For peer review only - http://bmjopen.bmj.com/site/about/guidelines.xhtml

69. Furber G, Segal L. The validity of the Child Health Utility instrument (CHU9D) as a routine outcome measure for use in child and adolescent mental health services. Health and Quality of Life Outcomes 2015;13(1):22.
70. Dillon CB, Fitzgerald AP, Kearney PM, et al. Number of Days Required to Estimate Habitual Activity Using Wrist-Worn GENEActiv Accelerometer: A Cross-Sectional Study. PLoS One 2016;11(5):e0109913.
71. Flick U. The SAGE Handbook of Qualitative Data Collection [online] 2018. https://sk.sagepub.com/reference/the-sage-handbook-of-qualitative-data-collection (Accessed 31st March 2023).
72. Galletta A. Mastering the Semi-Structured Interview and Beyond: From Research Design to Analysis and Publication. New York: New York University Press; 2013.
73. Dalglish SL, Khalid H, McMahon SA. Document analysis in health policy research: the READ approach. Health Policy and Planning 2020;35(10):1424-31.
74. Charles JM, Harrington DM, Davies MJ, et al. Micro-costing and a cost-consequence analysis of the 'Girls Active' programme: A cluster randomised controlled trial. PLoS One 2019;14(8):e0221276.
75. Stake RE. The art of case study research. Thousand Oaks, California: SAGE; 1995.
76. Braun V, Clarke V. Using thematic analysis in psychology. Qualitative Research in Psychology 2006;3(2):77-101.
77. Stake RE. Multiple case study analysis. New York: Guilford Press; 2006.
78. NIHR. Briefing notes for researchers- public involvement in NHS, health and social care research: NIHR [online] 2021. https://www.nihr.ac.uk/documents/briefing-notes-for-researchers-public-involvement-in-nhs-health-and-social-care-research/27371#who-should-i-involve (Accessed 23rd February 2022).
79. NIHR. Different experiences: A framework for consdiering who might be involved in research: NIHR [online] 2021. https://www.nihr.ac.uk/documents/different-experiences-a-framework-for-considering-who-might-be-involved-in-research/27387 (Accessed 23rd February 2022).
80. Staniszewska S, Brett J, Simera I, et al. GRIPP2 reporting checklists: tools to improve reporting of patient and public involvement in research. BMJ 2017;358:j3453.

For peer review only

FIGURE TITLE

Figure 1. Logic model and theory of change for the influence of school policies that restrict daytime use of smartphones on mental wellbeing and other associated behavioural and health outcomes in adolescents

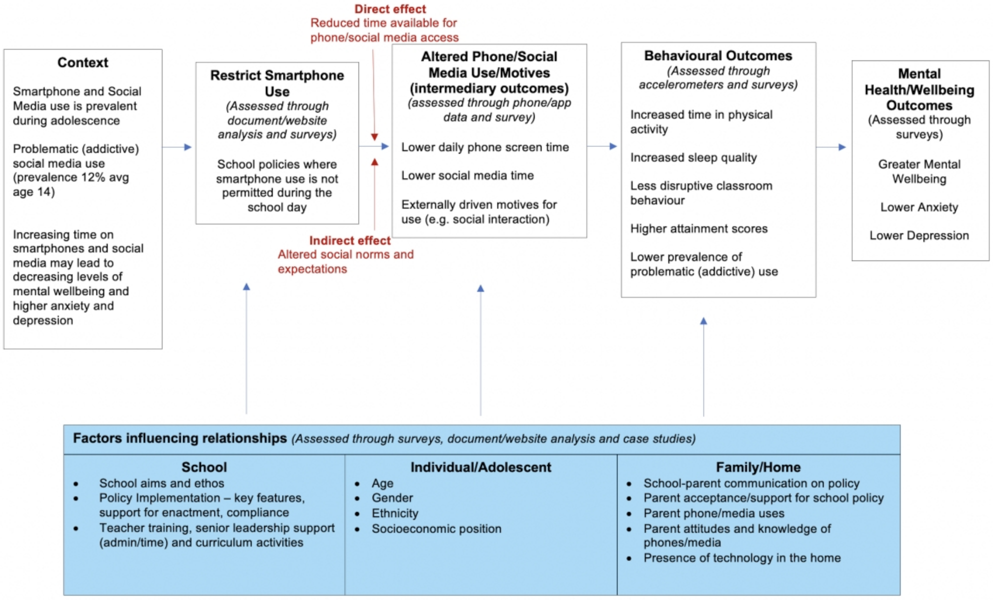

Figure 1. Logic model and theory of change for the influence of school policies that restrict daytime use of smartphones on mental wellbeing and other associated behavioural and health outcomes in adolescents.

411x248mm (300 x 300 DPI)

The TIDieR (Template for Intervention Description and Replication) Checklist*:

Information to include when describing an intervention and the location of the information

Item number	Item	Where located ** Primary paper (page or appendix number)	Other † (details)
	**BRIEF NAME**		
**1.**	*Name:* SMART Schools Study Smartphones, social Media and Adolescent mental wellbeing: the impact of school policies Restricting dayTime use<br>*Description:* The impact of school daytime restrictions of smartphone and social media use on adolescent mental wellbeing (primary outcome), anxiety, depression, physical activity, sleep, classroom behaviour, attainment, and addictive social media use.	P1, P6	P1 (NHS Health Research Authority (2023) SMART Schools Protocol V6 Available from: https://www.funding[illegible]wards.nihr.ac.uk/aw[illegible]d/NIHR131396)
	**WHY**		
**2.**	*Rationale:* Globally, mental disorders (e.g. anxiety and depression) are the leading cause of disability in adolescents (age range 10-19). At the same time, smartphone and social media use is prevalent during adolescence, and accounts for the majority of their overall screen time. At higher levels of use, time spent on smartphones and social media are associated with decreasing levels of mental wellbeing and higher levels of anxiety and depression.<br><br>There is considerable evidence that school-based interventions can have beneficial effects on adolescent mental wellbeing and associated behavioural outcomes (e.g., sleep, physical activity,	P4-9; Figure 1 on P7, Table 2 on P9	P1-5 (NHS Health Research Authority (2023) SMART Schools Protocol V6. Available from: https://www.fundingawards.nihr.ac.uk/award/NIHR131396)

For peer review only

classroom behaviour and attainment). School phone policies that restrict daytime phone/media use are an example of a current whole-school environment intervention.

Goal: We suggest that these policies have the potential to lower the overall time adolescents spend on the smartphones/social media, which may improve mental wellbeing and associated behavioural outcomes. There are variations in school-based policies, and these are classified as:

Table 2. Classifications of Variations in School Smartphone Policies as Restrictive (Intervention) and Permissive (Comparator) (P9).

Permissive School Phone Policies (Control)	Restrictive School Phone Policies (Intervention)
Allow pupils to carry phones on them and use them at any time point during the day	Allow phones onto school premises but insist these are not to be used during the school day and are turned off and out of site
Allow pupils to carry phones on them and use them at specific time points during the day (e.g. breaks and lunch)	Allow phones onto school premises, but only allow use of the phones if sanctioned by teaching staff for educational activities (e.g. use of calculator)
Allow pupils to carry phones on them and use them for personal use with consent from school staff	Allow phones onto premises but insist that phones are left in a particular place during the school day e.g. school reception
Allow pupils to carry phones on them and use within designated areas or zones	Pupils are not allowed to carry their phones on school premises all together

Theory: The intervention and data capture are directed by our logic model (Figure 1, P which integrates multiple theories and evidence. First, we adhere to displacement theories to propose that reducing the time adolescents spend on phones/media (i.e. restricting school time use) is optimal for mental wellbeing. Overuse can displace other mental wellbeing promoting activities (e.g. sleep and physical activity) and very low use can deprive adolescents of interactions that support mental wellbeing (e.g., affect and relationships). Second, psychological motives drive phone/media use; motives related to

For peer review only - http://bmjopen.bmj.com/site/about/guidelines.xhtml

	enhancement and social interactions promote mental wellbeing, and motives related to coping and conformity (e.g. Fear of Missing Out) are associated with problematic use (addiction) and poor mental wellbeing. Hence the school policy and ethos have the potential to influence adolescents' motives for using phones/media, which may impact on mental wellbeing. Third, the ecological model of social influence proposes three agents that shape wellbeing and technology use, including the school environment, home/family and individual factors. Finally, policy enactment and implementation process models identify that school policy implementation effects will be shaped by social processes (e.g., training, leadership, compliance, administration, family-school interactions).		
	**WHAT**		
**3.**	*Materials:* Materials for the intervention are context dependent and will vary across schools implementing a restrictive phone policy. Based on our PPI activities, materials may include policy documents; policy communication with parents and adolescents e.g. through school information packs, assemblies, letters and/or the school website.	P7, Table 1 on P8	P7-8, Table 1 on P9 (NHS Health Research Authority (2023) SMART Schools Protocol V6. Available from: https://www.fundingawards.nihr.ac.uk/award/NIHR131396)
**4.**	*Procedures:* Adolescents are not permitted to use their smartphones during lessons or recreational time in the school day, and their smartphones must not be seen in school during these times (except with permission from a teacher)	Table 2 on P9	Table 2 on P10 (NHS Health Research Authority (2023) SMART Schools Protocol V6. Available from: https://www.fundingawards.nihr.ac.uk/award/NIHR131396)

For peer review only - http://bmjopen.bmj.com/site/about/guidelines.xhtml

	**WHO PROVIDED**		
**5.**	Schools develop their own policies (or these are designed by the school's Multi-Academy Trust), and this policy is communicated to all school staff and pupils. Secondary Schools within the sample (see point 7) vary in regions of high and low levels of deprivation and in areas that have high and low proportions of Black, Asian and Minority Ethnic (BAME) groups.	Types of policies presented in Table 2 on P9	Table 2 on P10 (NHS Health Research Authority (2023) SMART Schools Protocol V6. Available from: https://www.fundingawards.nihr.ac.uk/award/NIHR131396)
	**HOW**		
**6.**	*Mode of delivery:* The restrictive school policy is implemented in school contexts, and thus, a face-to-face format. School staff enact the school policy and, in some schools, are required to administer behavioural consequences for adolescents who use their phone during the day, such as: phone confiscation, detention, parent-school meeting. In the intervention schools the restrictive school policy is applied to pupils in year groups 7-11 (age 11-16 years).	Table 2 on P9, Table 3 on P12	Table 1 on P9, Table 2 on P10 (NHS Health Research Authority (2023) SMART Schools Protocol V6. Available from: https://www.fundingawards.nihr.ac.uk/award/NIHR131396)
	**WHERE**		
**7.**	*Where the intervention occurred:* Secondary schools (ages 11-19) within England and located within a 100-mile radius of the recruiting centre in the West Midlands. 64 local authorities are included in the sampling frame from the West Midlands, East Midlands, East, South East, South West and North West. Secondary schools in the sampling frame (including community schools, foundation and voluntary schools, academies, free schools and colleges), had Senior Leadership Team members, school staff and parents/carers capable of giving informed consent, and school pupils aged 12-13 or 14-15 capable of providing assent were included.	P9-11	P12 (NHS Health Research Authority (2023) SMART Schools Protocol V6. Available from: https://www.fundingawards.nihr.ac.uk/award/NIHR131396)

For peer review only - http://bmjopen.bmj.com/site/about/guidelines.xhtml

	Schools other than state-funded mainstream schools (special schools, pupil referral units and independent schools) were excluded because it was expected that there would be additional influences on mental wellbeing. Schools that did not have an accessible smartphone policy and/or had different smartphone policies for the year groups 8 and 10, and schools with missing data that was required for the propensity score estimation were also excluded.		
**8.**	**WHEN and HOW MUCH**<br>Intervention schools in the sample will already have a restrictive policy implemented, hence this study is a natural experiment and observational study. Intervention schools vary in the level of restrictions for phone/media use, with some restrictive school policies permitting pupil phones to be placed in their bags, whereas others do not permit pupils to bring their phones onto the school premises. Intervention schools vary in the duration that the school phone/media policy has been implemented (e.g. <1, 1, 2, or 3> years).	P6-9, Table 2 on P9	P10 (NHS Health Research Authority (2023) SMART Schools Protocol V6. Available from: https://www.fundingawards.nihr.ac.uk/award/NIHR131396)
**9.**	**TAILORING**<br>Schools have developed policies that are tailored to their specific school contexts (or Multi-Academy Trusts). Policies usually apply to the whole school, although in some schools, 6th form pupils (age 16+) may be permitted to use their smartphones during the school day (this age group will not be investigated in this study).	Table 1 on P8,<br>Table 2 on P9	Table 2 on P10 (NHS Health Research Authority (2023) SMART Schools Protocol V6. Available from: https://www.fundingawards.nihr.ac.uk/award/NIHR131396)
**10.**	**MODIFICATIONS**	N/A	

For peer review only

For peer review only - http://bmjopen.bmj.com/site/about/guidelines.xhtml

	N/A		
**11.**	**HOW WELL**<br>Adherence: Adherence of the intervention will be captured by the degree to which pupils, school staff and parents adhere to the school policy, and how parents/carers support of the policy varies across schools.	Table 1 on P8, Table 3 on P12	P11 (NHS Health Research Authority (2023) SMART Schools Protocol V6. Available from: https://www.fundingawards.nihr.ac.uk/award/NIHR131396)
**12.**	Actual: N/A	N/A	N/A

** **Authors** - use N/A if an item is not applicable for the intervention being described. **Reviewers** – use '?' if information about the element is not reported/not sufficiently reported.

† If the information is not provided in the primary paper, give details of where this information is available. This may include locations such as a published protocol or other published papers (provide citation details) or a website (provide the URL).

‡ If completing the TIDieR checklist for a protocol, these items are not relevant to the protocol and cannot be described until the study is complete.

* We strongly recommend using this checklist in conjunction with the TIDieR guide (see *BMJ* 2014;348:g1687) which contains an explanation and elaboration for each item.

* The focus of TIDieR is on reporting details of the intervention elements (and where relevant, comparison elements) of a study. Other elements and methodological features of studies are covered by other reporting statements and checklists and have not been duplicated as part of the TIDieR checklist. When a **randomised trial** is being reported, the TIDieR checklist should be used in conjunction with the CONSORT statement (see www.consort-statement.org) as an extension of **Item 5 of the CONSORT 2010 Statement.** When a **clinical trial protocol** is being reported, the TIDieR checklist should be used in conjunction with the SPIRIT statement as an extension of **Item 11 of the SPIRIT 2013 Statement** (see www.spirit-statement.org). For alternate study designs, TIDieR can be used in conjunction with the appropriate checklist for that study design (see www.equator-network.org).

For peer review only

For peer review only - http://bmjopen.bmj.com/site/about/guidelines.xhtml
