# Supplementary material for: Smartphones, social Media and Adolescent mental well-being: the impact of school policies Restricting dayTime use—protocol for a natural experimental observational study using mixed methods at secondary schools in England (SMART Schools Study)
Source: BMJ Open. 2023 Jul 5;13(7):e075832. doi: 10.1136/bmjopen-2023-075832 (PMC10335462; doi:10.1136/bmjopen-2023-075832)
Supplement: Reviewer comments [file bmjopen-2023-075832.reviewer_comments.pdf]

PEER REVIEW HISTORY

BMJ Open publishes all reviews undertaken for accepted manuscripts. Reviewers are asked to complete a checklist review form (**http://bmjopen.bmj.com/site/about/resources/checklist.pdf**) and are provided with free text boxes to elaborate on their assessment. These free text comments are reproduced below.

ARTICLE DETAILS

**TITLE (PROVISIONAL)**	Smartphones, social Media and Adolescent mental wellbeing: the impact of school policies Restricting dayTime use—protocol for a natural experimental observational study using mixed methods at secondary schools in England (SMART Schools Study)
**AUTHORS**	Wood, Grace; Goodyear, Victoria; Adab, Peymane; Al-Janabi, Hareth; Fenton, Sally; Jones, Kirsty; Michail, Maria; Morrison, Breanna; Patterson, Paul; Sitch, Alice; Wade, Matthew; Pallan, Miranda

This article was not externally reviewed at BMJ Open. Protocols that have been independently assessed prior to submission to BMJ Open are usually fast-tracked to publication on the grounds that further substantial changes will not be possible. This independent assessment will usually be external, independent review for both a substantial grant award from a non-commercial or government funder and ethics approval.
